# GSNOR reprograms nitrosylation to drive endothelial-to-mesenchymal transition and fibrotic vascular remodeling

**DOI:** 10.1016/j.redox.2026.104339

**Published:** 2026-08-07

**Authors:** Zhimin Song, Yun Zhang, Jingjing Chen, Xing Peng, Jiaying Fan, Yao Pan, Xiuxia Yang, Tinghong Zhang, Hui Zheng, Shu Xia, Qun Luo, Bin Zhou, Shu Meng

**Affiliations:** a State Key Laboratory of Respiratory Disease, the First Affiliated Hospital, Guangzhou Medical University, Guangzhou, Guangdong, 510120, China; b Department of Basic Science Research, Guangzhou National Laboratory, Guangzhou, Guangdong, 510005, China; c Department of Ophthalmology, the Fifth Affiliated Hospital of Sun Yat-sen University, Zhuhai, Guangdong, 519000, China; d CAS CEMCS-CUHK Joint Laboratories, New Cornerstone Science Laboratory, Key Laboratory of Multi-Cell Systems, Shanghai Institute of Biochemistry and Cell Biology, Center for Excellence in Molecular Cell Science, Chinese Academy of Sciences, University of Chinese Academy of Sciences, Shanghai, 200031, China

**Keywords:** GSNOR, EndoMT, Pulmonary fibrosis, Denitrosylation, HMGB1

## Abstract

Endothelial cells are central regulators of tissue regeneration, yet how redox signaling governs the balance between vascular repair and fibrosis remains incompletely understood. Here, we identify S-nitrosoglutathione reductase (GSNOR), a key enzyme controlling S-nitrosoglutathione metabolism and protein S-nitrosylation, as a critical regulator of endothelial fate in idiopathic pulmonary fibrosis (IPF). GSNOR expression is markedly elevated in the pulmonary endothelium of IPF patients and correlates with disease severity, indicating disrupted S-nitrosothiol homeostasis during fibrogenesis. Genetic or pharmacologic inhibition of GSNOR, either globally or in endothelial cells, restores S-nitrosylation balance, preserves endothelial identity, and protects against fibrosis. In contrast, endothelial-specific GSNOR overexpression enhances denitrosylation, promoting endothelial-to-mesenchymal transition (EndoMT) and exacerbating fibrotic remodeling. Quantitative S-nitrosoproteomic analysis reveals that GSNOR broadly remodels S-nitrosylation networks governing extracellular matrix organization and endothelial signaling. Mechanistically, reduced high mobility group box 1 (HMGB1) Cysteine-23 S-nitrosylation facilitates its cytoplasmic translocation and potentiates TGF-β–driven EndoMT, linking GSNOR-associated S-nitrosylation remodeling to fibrogenic endothelial reprogramming. Collectively, these findings define endothelial GSNOR as a key regulator of S-nitrosylation-dependent endothelial plasticity and fibrotic vascular remodeling.

## Highlights

• GSNOR regulates endothelial S-nitrosylation during pulmonary fibrosis.
• HMGB1 Cys23 denitrosylation promotes HMGB1 cytoplasmic translocation.
• GSNOR-driven HMGB1 translocation links redox imbalance to endothelial remodeling.
• Targeting GSNOR suppresses EndoMT and attenuates lung fibrosis.

## 1 Introduction

Endothelial cells (ECs), which line the inner surface of blood vessels, are central regulators of vascular homeostasis and angiogenic regeneration [1]. Following injury, sufficient endothelial proliferation supports angiogenesis and regeneration, whereas aberrant endothelial-to-mesenchymal transition (EndoMT) diverts repair toward fibrosis [2]. In the EndoMT process, ECs lose lineage markers, acquire mesenchymal traits, and compromise vascular integrity. Despite extensive investigation into EC plasticity, the upstream signals that govern this regenerative versus fibrotic bifurcation remain poorly defined.

Among these potential regulators, nitric oxide (NO) signaling stands out as a key determinant of endothelial fate. NO promotes endothelial survival, proliferation, and angiogenic regeneration [3], acting in part through protein S-nitrosylation [4], a reversible redox modification in which a NO group covalently attaches to reactive cysteine residues. NO signaling also strongly antagonizes EndoMT [5]. S-nitrosoglutathione reductase (GSNOR) is a central negative regulator of NO signaling. It metabolizes GSNO to GSH and controls intracellular S-nitrosylation homeostasis through the trans-nitrosylation between GSNO and protein-SNO [6,7]. Yet whether GSNOR actively reprograms endothelial S-nitrosylation to drive EndoMT and fibrosis remains unexplored.

This question bears particular significance for idiopathic pulmonary fibrosis (IPF), a devastating interstitial lung disease characterized by relentless fibroblast activation, extracellular matrix deposition, and vascular remodeling [[8], [9], [10]]. Despite antifibrotic therapies [11,12], survival remains limited to 3–5 years after diagnosis, and lung transplantation is the only curative option. Increasing evidence implicates EndoMT as a source of profibrotic cells in IPF [[13], [14], [15], [16]], raising the possibility that blocking EndoMT may offer therapeutic benefit.

Here, we identify endothelial GSNOR as a molecular switch that governs endothelial fate determination in a lung fibrosis model. We demonstrate that aberrant GSNOR upregulation reshapes the S-nitrosylation landscape, promotes EndoMT, and contributes to fibrotic vascular remodeling through reducing HMGB1 Cysteine-23 (C23) S-nitrosylation. These findings uncover the GSNOR-protein SNO axis as a central determinant of endothelial reprogramming and suggest that targeting GSNOR may offer a promising therapeutic strategy for fibrotic lung disease.

## 2 Results

### 2.1 GSNOR upregulation in ECs correlates with reduced GSNO levels in pulmonary fibrosis

**Fig. 1:**
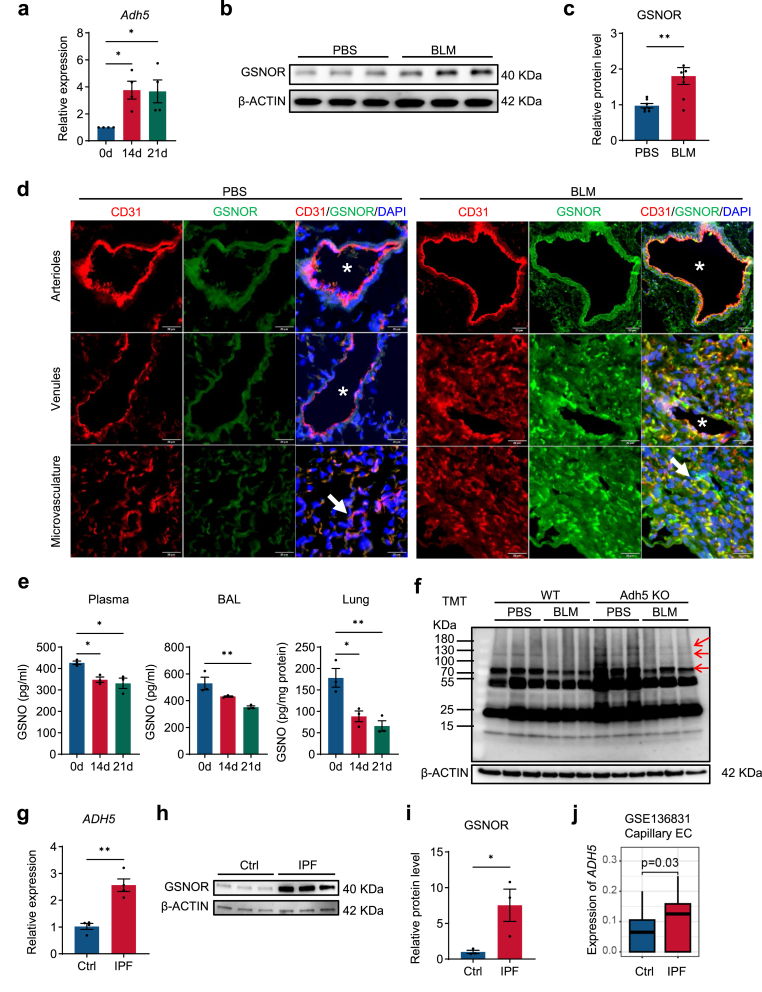
Elevated GSNOR expression in murine and human pulmonary fibrosis. (a) *Adh5* gene levels in lung tissues of WT mice are significantly increased at 14 and 21 days post-BLM challenge. n = 4 mice per group, mean ± SEM. *P < 0.05, one-way ANOVA. (b) Representative immunoblots for GSNOR in lung homogenates from WT mice treated with BLM or PBS for 21 days. n = 3 mice per group. (c) Densitometric analysis of GSNOR protein levels in WT mice treated with BLM or PBS for 21 days. Data are presented as mean ± SEM; each dot represents one mouse; n ≥ 7 mice per group from two independent experiments. ****P < 0.0001, Student's t-test. (d) Immunofluorescence staining of lung sections with anti-CD31, anti-GSNOR, and DAPI revealed elevated GSNOR expression in ECs. Scale bar = 20 μm. (e) GSNO levels are significantly decreased in plasma, BAL fluid, and lung homogenates from BLM-treated mice, determined by ELISA. n = 3 mice per group, mean ± SEM. *P < 0.05, **P < 0.01, one-way ANOVA. (f) Global protein S-nitrosylation was assessed by an iodo-TMT switch assay without enrichment. After blocking free thiols, S-nitrosylated cysteines were selectively reduced, labeled with iodo-TMT, and detected in total lung lysates by anti-TMT Western blot. Iodo-TMT-labeled SNO protein signals, particularly those above 70 kDa, were decreased 21 days after BLM challenge. (g) *ADH5* gene levels in lung tissues are significantly elevated in IPF patients compared to non-IPF donors. Data are presented as mean ± SEM; each dot represents one donor or patient; n = 4 independent human samples per group. P < 0.01, Student's t-test. (h) Representative immunoblots showing increased GSNOR protein levels in lung homogenates from IPF patients compared to non-IPF (Ctrl) donors. n = 3 independent human samples per group. (i) Densitometric analysis of GSNOR protein levels. Data are presented as mean ± SEM; each dot represents one donor or patient; n = 3 independent human samples per group. P < 0.05, Student's t-test. (j) Higher average GSNOR expression in ECs of IPF patients compared to non-IPF donors, based on data from GSE136831, IPF Cell Atlas.

We investigated the expression and functional implications of GSNOR in both a murine model of BLM-induced pulmonary fibrosis and human IPF tissues. Our results revealed that GSNOR was significantly and specifically upregulated in pulmonary ECs during fibrosis. In the murine model, the levels of *Adh5* mRNA and its encoded protein GSNOR were significantly elevated at days 14 and 21 post-BLM (Fig. 1a–c). This upregulation was predominantly observed in arteriolar, venular, and microvascular ECs, as confirmed by CD31 immunostaining (Fig. 1d), indicating a spatially restricted elevation of GSNOR expression within the pulmonary vasculature.

A critical question is whether GSNOR's upregulation in ECs influences the global protein-SNO level through denitrosylation of GSNO. Our data showed that GSNO levels, the substrate for GSNOR, were significantly reduced in bronchoalveolar lavage (BAL) fluid, lung homogenates, and plasma (Fig. 1e), indicating that elevated GSNOR activity reduced both local pulmonary and systemic GSNO levels. Additionally, immunoblotting showed a marked decrease in SNO-proteins above 70 kDa at 21 days post-BLM, compared to controls, as detected by iodo-TMT labeling (Fig. 1f) or anti-SNO antibodies (Fig. S1). While these blot-based assays provide an overview of global S-nitrosylation changes, they are inherently semi-quantitative and can be influenced by differences in protein abundance. Therefore, we next performed quantitative site-resolved LC–MS/MS analysis to comprehensively characterize alterations in protein S-nitrosylation.

In IPF patient samples, we observed consistent upregulation of GSNOR at both the gene and protein levels compared to non-IPF donors (Fig. 1g–i). Analysis of two scRNA-seq datasets [16,17] from two independent IPF patient cohorts confirmed increased ADH5 gene expression specifically in capillary ECs, further validating our observations in human tissues (Fig. 1j and Fig. S2a–b).

### 2.2 EndoMT-associated changes occur during BLM-induced murine pulmonary fibrosis and in human IPF tissues

**Fig. 2:**
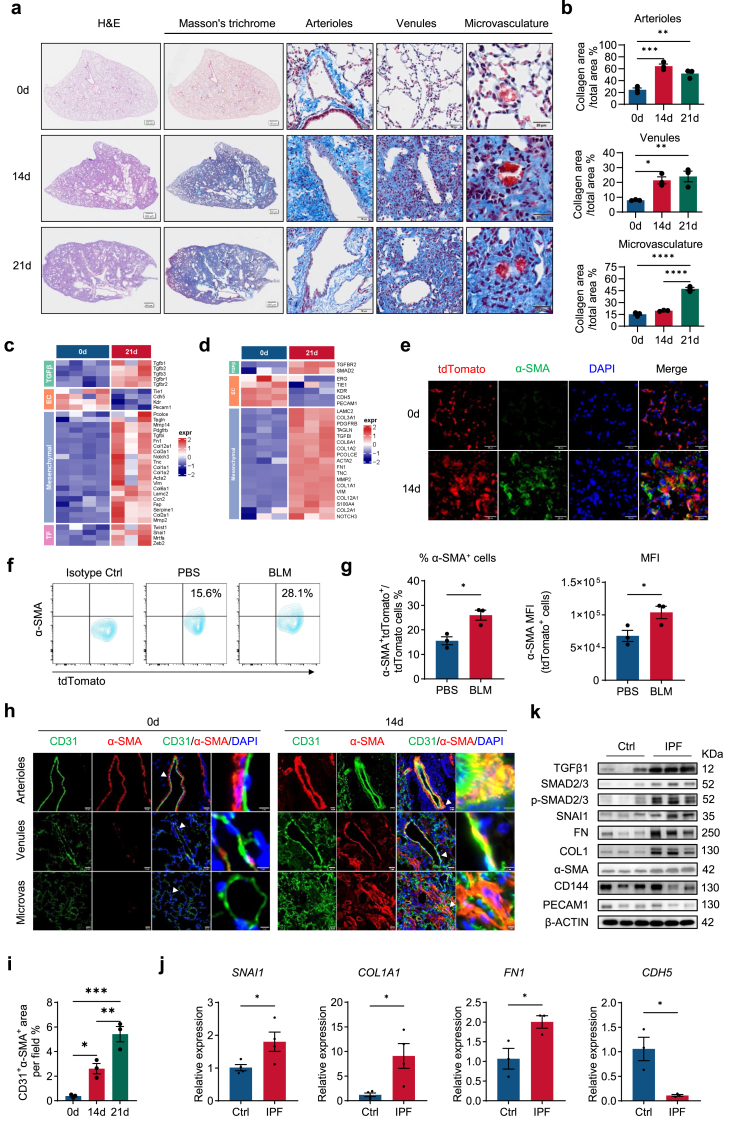
Profiling EndoMT in mouse models and human tissues. (a) Histological analysis with H&E and Masson's trichrome staining demonstrates increased cellularity and collagen accumulation around arterioles, venules, and microvasculature in BLM-treated mice. (b) Quantification of collagen-positive area relative to total lung area based on Masson's trichrome staining. For each sample, three randomly selected fields were quantified and averaged to generate one biological value. Data are presented as mean ± SEM; n = 3 mice per group. *P < 0.05, **P < 0.01, ***P < 0.001, ****P < 0.0001, one-way ANOVA. (c) RNA sequencing reveals increased TGF-β signaling, mesenchymal marker expression, and transcription factors related to EndoMT, along with decreased EC gene expression in BLM-treated mice at 21 days post-challenge. n ≥ 3 mice per group. (d) Mass spectrometry at 21 days post-challenge shows increased TGF-β signaling and mesenchymal marker expression and decreased EC gene expression. n = 3 independent biological samples per group. (e) Immunofluorescence shows co-expression of tdTomato and α-SMA, indicating increased EndoMT in BLM-treated Cdh5-creERT2; *R26R*-tdTomato mice at 14 days. Scale bar = 20 μm. (f) Flow cytometry shows increased α-SMA expression in tdTomato^+^ cells in the lungs of BLM-treated Cdh5-creERT2; *R26R*-tdTomato mice compared to PBS controls, indicating enhanced EndoMT in lineage-traced ECs. (g) Percentage of α-SMA^+^ tdTomato^+^ cells and mean fluorescence intensity (MFI) of tdTomato^+^ cells in lungs of PBS- or BLM-treated Cdh5-creERT2; *R26R*-tdTomato mice at 14 days, as measured by flow cytometry. n = 3 mice per group, mean ± SEM. *P < 0.05, Student's t-test. (h) Immunofluorescence staining shows co-expression of CD31 and α-SMA, indicating significant increases in EndoMT in BLM-treated mice. Scale bar: 20 μm; 5 μm in the last merged panel. (i) Quantification of the percentage of CD31 and α-SMA co-positive area. Fifteen randomly selected fields from three independent mice were quantified, with five fields analyzed per mouse. The five field-level values from each mouse were averaged to generate one biological replicate. Data are presented as mean ± SEM; n = 3 mice per group. *P < 0.05, **P < 0.01, ***P < 0.001, one-way ANOVA. (j) qPCR analysis in lung tissues from IPF patients and non-IPF donors showing significant increases in *SNAI1*, *COL1A1*, and *FN1* expression and a significant decrease in *CDH5* expression in IPF patients. n ≥ 3 independent human samples per group., means ± SEM. *P < 0.05, Student's t-test. (k) Western blot analysis in lung tissues from IPF patients and non-IPF donors, showing TGF-β pathway proteins (TGF-β1, SMAD2/3, phospho-SMAD2/3, SNAIl) and mesenchymal markers (FN, COL1, α-SMA), and endothelial markers PECAM1 and CD144. n = 3 independent human samples per group.

We next examined whether EndoMT-associated changes occur during fibrotic lung remodeling. Histological analysis using H&E and Masson's trichrome staining showed increased cellularity and diffuse collagen deposition in BLM-treated mice compared with controls, with fibrotic changes observed in both perivascular and interstitial regions (Fig. 2a and b). RNA-seq and proteomic analysis by mass spectrometry revealed elevated TGFβ signaling, mesenchymal markers, and fibrotic transcription factors alongside downregulated endothelial markers during pulmonary fibrosis development (Fig. 2c and d). These findings were validated by qPCR and Western blot analyses, which showed elevated expression of *Snai1, Acta2, Col1a1,* and *Fn1,* coupled with increased protein levels of SNAIl and α-SMA, and reduced PECAM1 expression in BLM-treated lung tissues at 14 and 21 days post-challenge (Fig. S2c–e).

To confirm the occurrence of EndoMT *in vivo*, we utilized *Cdh5-*creERT2; *R26R-*tdTomato lineage-tracing mice, in which ECs were permanently labeled with tdTomato upon tamoxifen induction. Post-BLM treatment, we observed a significant increase in α-SMA expression within tdTomato^+^ cells, indicative of EndoMT, as examined by microscopy (Fig. 2e) and flow cytometry (Fig. 2f and g). In WT mice, under homeostatic conditions, CD31 immunofluorescence was restricted to vascular ECs and α-SMA to vascular smooth muscle cells. However, following BLM treatment, CD31^+^ ECs displayed increased α-SMA expression across all vessel types, especially in the microvasculature (Fig. 2h and i), further supporting EndoMT induction.

We further performed molecular analyses in tdTomato ^+^ cells isolated from lung tissues from *Cdh5*-creERT2; *R26R*-tdTomato mice treated with either PBS or BLM. In BLM-treated mice, *Adh5* expression was significantly upregulated in tdTomato ^+^ cells, along with robust increases in mesenchymal markers such as *Snai1* and *Col1a1* compared to PBS-treated controls (Fig. S2f).

Consistent with these findings, lung tissues from IPF patients exhibited similar molecular and histological evidence of EndoMT. RT-qPCR analysis revealed significantly increased expression of *SNAI1, COL1A1,* and *FN1,* along with reduced *CDH5* levels compared to controls (Fig. 2j). Protein analyses further validated these findings, showing elevated levels of TGF-β signaling components (TGF-β1, SMAD2/3, phospho-SMAD2/3, SNAI1) and mesenchymal proteins (Fibronectin (FN), Collagen 1 (COL1), α-SMA). Although PECAM1 and CD144 levels were not significantly different, their overall expression was reduced in IPF patients compared to controls (Fig. 2k and Fig. S2g).

### 2.3 GSNOR mediates EndoMT *in vitro* and *in vivo*

To elucidate the role of GSNOR in EndoMT, we employed a TGF-β-induced EndoMT model in human pulmonary microvascular endothelial cells (HPMECs) to mimic the *in vivo* EndoMT process during lung fibrosis.

TGF-β2 treatment effectively induced EndoMT, as evidenced by increased expression of key transcription factors *SNAI1* and mesenchymal-related genes (*ACTA2*, *SM22*, *VIM*, *FN1*, *COL1A1*). Endothelial markers, including *PECAM1*, were modestly reduced (Fig. S3a). Western blot confirmed elevated levels of SNAIl and FN at both 6 and 24 h post-TGF-β2 stimulation (Fig. S3b–e). Given its robust upregulation at both gene and protein levels, we selected FN as a marker for subsequent EndoMT analysis.

**Fig. 3:**
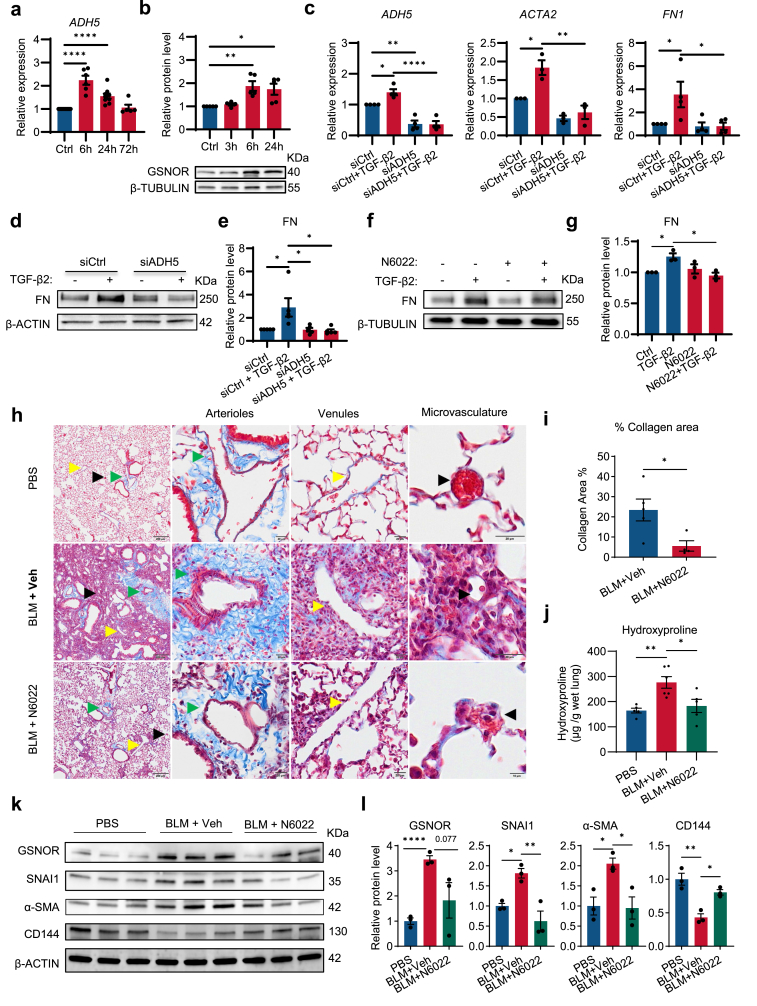
GSNOR promotes EndoMT both *in vitro* and *in vivo*. (a) *ADH5* gene expression was significantly increased in HPMECs at 6 and 24 h after stimulation with TGF-β2 (10 ng/mL). Data are presented as mean ± SEM; n ≥ 5 independent biological replicates per group. ****P < 0.0001, one-way ANOVA. (b) Upper, densitometric quantification of Western blot analysis showing increased GSNOR protein levels in HPMECs at 6 and 24 h after stimulation with TGF-β2 (10 ng/mL). Protein levels were normalized to the corresponding loading control. Data are presented as mean ± SEM; n = 5 independent biological replicates per group. *P < 0.05, **P < 0.01, one-way ANOVA. Lower, representative Western blots. (c) *ADH5* knockdown by siRNA reduced ACTA2 and FN1 gene expression in HPMECs after stimulation with TGF-β2 (10 ng/mL). Data are presented as mean ± SEM; n ≥ 3 independent biological replicates per group. *P < 0.05, **P < 0.01, ****P < 0.0001, one-way ANOVA. (d, e) Representative Western blot images (d) and densitometric quantification (e) showing decreased FN protein levels in *ADH5*-knockdown HPMECs after stimulation with TGF-β2 (10 ng/mL). Protein levels were normalized to the corresponding loading control. Data are presented as mean ± SEM; n = 5 independent biological replicates per group. *P < 0.05, one-way ANOVA. (f, g) Representative Western blot images (f) and densitometric quantification (g) showing decreased FN protein levels in HPMECs treated with N6022 (1 μM) after stimulation with TGF-β2 (10 ng/mL). Protein levels were normalized to the corresponding loading control. Data are presented as mean ± SEM; n = 3 independent biological replicates per group. *P < 0.05, one-way ANOVA. (h) Masson's trichrome staining of lung sections showing reduced collagen deposition in N6022-treated mice (30 mg/kg) compared to vehicle-treated controls after 14 days of BLM challenge. A PBS-treated control is included for comparison. (i) Quantification of collagen-positive area relative to total lung area in vehicle- and N6022-treated mice after BLM challenge for 14 days. N6022 was administered at 30 mg/kg. Data are presented as mean ± SEM; n ≥ 4 mice per group. *P < 0.05, Student's t-test. (j) Quantification of hydroxyproline levels in lung tissue using hydroxyproline assay kits, showing reduced collagen content in N6022-treated mice (30 mg/kg) compared to vehicle controls after 14 days of BLM challenge. A PBS-treated control is included for reference. n ≥ 5 mice per group, mean ± SEM. *P < 0.05, **P < 0.01, one-way ANOVA. (k) Western blot analysis showing decreased SNAI1 and α-SMA expression and increased CD144 expression in 30 mg/kg N6022-treated mice compared to vehicle controls after 14 days of BLM challenge. n = 3 mice per group. (l) Densitometric analysis of GSNOR, SNAIl, CD144, and α-SMA protein levels. n = 3 mice per group, mean ± SEM. *P < 0.05, **P < 0.01, ***P < 0.001, ****P < 0.0001, one-way ANOVA.

TGF-β2 stimulation led to a transient but significant upregulation of *ADH5* gene and protein expression at 6 and 24 h, which returned to baseline levels by 72 h (Fig. 3a and b). To assess whether GSNOR directly mediates TGF-β-induced EndoMT, we performed genetic and pharmacologic inhibition experiments. Knockdown of *ADH5* using siRNA reduced GSNOR gene expression to 20% and protein expression to 30% of control levels (Fig. S3f–h). This significantly inhibited EndoMT, as indicated by reduced *ACTA2* expression at the gene level and *FN1* expression at both the gene and protein levels (Fig. 3c–e). Similarly, treatment with the GSNOR inhibitor N6022 produced comparable reductions in FN expression (Fig. 3f and g). These findings establish GSNOR as a critical regulator of TGF-β-induced EndoMT.

To investigate the role of GSNOR in pulmonary fibrosis, we treated mice with N6022 prior to BLM challenge (Fig. S4a). Masson's trichrome staining revealed significant reductions in collagen deposition in lung tissues of N6022-treated mice compared to vehicle controls (Fig. 3h and i), and this was corroborated by decreased hydroxyproline levels, a quantitative marker of collagen content (Fig. 3j). EndoMT markers were also evaluated *in vivo*. N6022 treatment significantly reduced the expression of GSNOR, SNAI1*,* and α-SMA while enhancing CD144 levels (Fig. 3k and l). These findings indicate that GSNOR inhibition attenuates EndoMT and its downstream contribution to fibrosis.

Given GSNOR's role in inflammation, we examined the inflammatory cell composition in BAL fluid using flow cytometry (Fig. S4b). Following BLM administration, there was a significant increase in inflammatory cell populations. N6022 treatment increased the percentage of alveolar macrophages (AMs) while reducing inflammatory cells, including neutrophils, T cells, and B cells. Importantly, total BAL leukocyte counts, elevated by BLM treatment, were significantly reduced by N6022 at a 30 mg/kg dose, although levels remained above those of PBS-treated controls (Fig. S4c–d).

To validate these findings, we administered another GSNOR inhibitor, N91115, orally (Fig. S5a). Similar to N6022, N91115 reduced BAL leukocyte counts, particularly AMs and pro-inflammatory monocytes (Fig. S5b–c). Additionally, N91115 treatment decreased collagen deposition (Fig. S5d–e) and hydroxyproline levels (Fig. S5f). Protein analysis confirmed reduced levels of GSNOR, SNAI1, and α-SMA, along with increased CD144 expression (Fig. S5g–h). These data further support the role of GSNOR in mediating both EndoMT and fibrosis.

### 2.4 Genetic deletion of *Adh5* attenuates EndoMT and pulmonary fibrosis

To further elucidate the regulatory role of GSNOR in EndoMT and pulmonary fibrosis *in vivo*, we utilized *Adh5* KO mice. *Adh5* KO was validated by genotyping and protein analysis (Fig. S6a–d).

EndoMT was significantly suppressed in *Adh5* KO mice, as evidenced by reduced expression of key mesenchymal markers such as SNAI1 and α-SMA, coupled with an increase in endothelial marker CD144 levels (Fig. S6d–e). Pulmonary fibrosis was markedly attenuated in *Adh5* KO mice. Masson's trichrome staining of lung tissues revealed reduced collagen deposition in the parenchyma (Fig. S6f–g), while hydroxyproline assays showed a significant decrease in total collagen content (Fig. S6h). BAL fluid analysis further revealed a dampened inflammatory response in *Adh5* KO mice. Total leukocyte counts were significantly reduced in the absence of GSNOR, with notable decreases in AMs, neutrophils, and B cells at 14 days post-BLM treatment compared to WT littermate controls (Fig. S6i–j).

### 2.5 EC-specific deletion of *Adh5* attenuates EndoMT, lung function, and pulmonary fibrosis

**Fig. 4:**
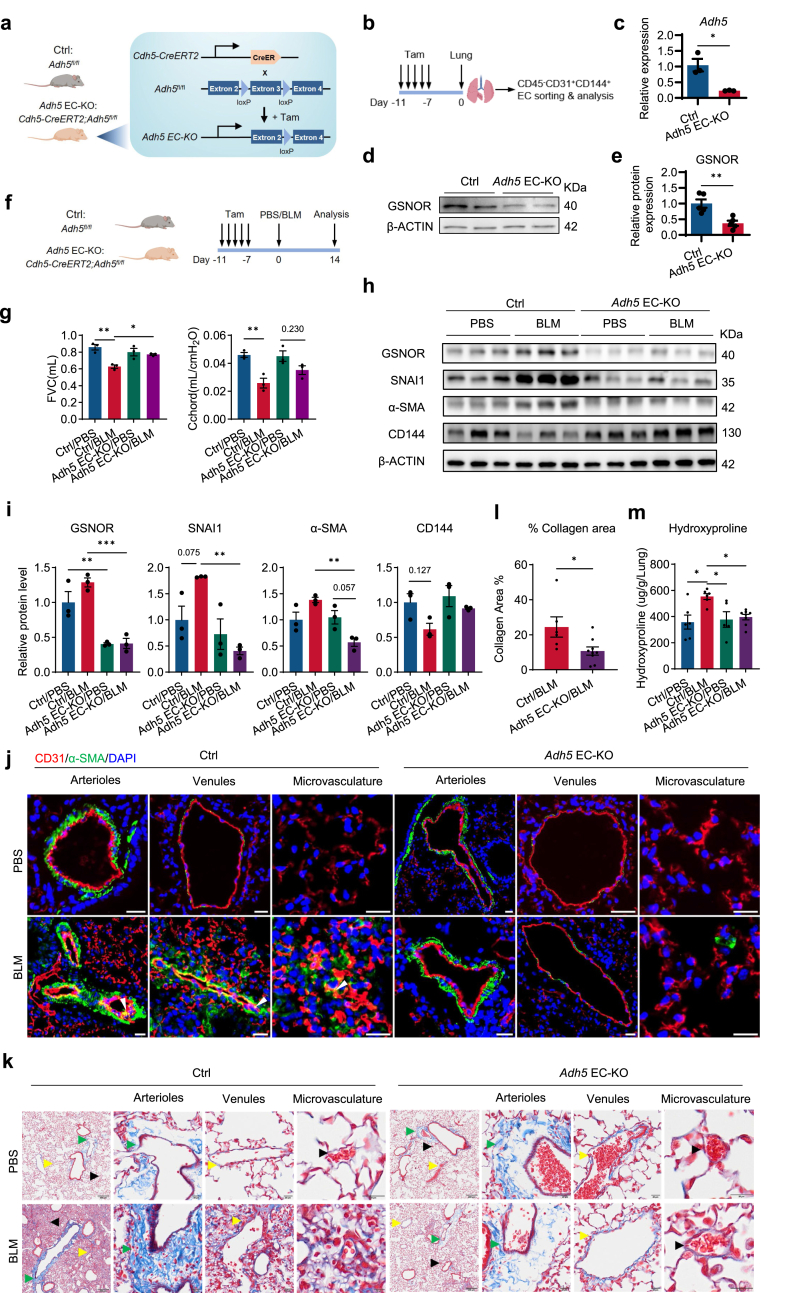
EC-specific Adh5 deletion attenuates EndoMT and bleomycin-induced pulmonary fibrosis. (a) Schematic representation of the CRISPR/Cas9 targeting strategy used to generate EC-specific *Adh5* knockout mice, showing Cre-mediated deletion of exon 3. *Adh5* EC-KO mice were defined as *Cdh5*-CreERT2;*Adh5*^*fl/fl*^ mice, and *Adh5*^*fl/fl*^ littermate mice were used as controls. (b) Schematic of tamoxifen induction and lung EC sorting. *Adh5* EC-KO mice and *Adh5*^*fl/fl*^ littermate controls received intraperitoneal injections of tamoxifen (100 mg/kg) for 5 consecutive days, followed by a 1-week waiting period to allow *Adh5* deletion in ECs. Lung ECs were sorted by flow cytometry as CD31^+^CD144^+^ cells. (c) qPCR analysis of Adh5 expression in sorted lung ECs from control and *Adh5* EC-KO mice. Data are presented as mean ± SEM; n = 3 mice per group. *P < 0.05, Student's t-test. (d) Representative Western blot showing reduced GSNOR protein levels in sorted lung ECs from *Adh5* EC-KO mice compared with controls. n = 2 mice per group. (e) Densitometric quantification of GSNOR protein levels in sorted lung ECs. Protein levels were normalized to the corresponding loading control. Data are presented as mean ± SEM; n = 5 mice per group. **P < 0.01, Student's t-test. (f) Schematic of the bleomycin-induced lung fibrosis model. Mice were administered bleomycin intratracheally at 2.5 U/kg, and lung samples were collected 14 days post-challenge. (g) Lung function analysis of FVC and Cchord. Data are presented as mean ± SEM; n = 3 mice per group. *P < 0.05, **P < 0.01, one-way ANOVA. (h) Western blot analysis showing decreased SNAI1 and α-SMA expression and increased CD144 expression in *Adh5* EC-KO mice compared to littermate controls at 14 days post-BLM challenge. n = 3 mice per group. (i) Densitometric quantification of GSNOR, SNAI1, CD144, and α-SMA protein levels in lung tissue. Protein levels were normalized to the corresponding loading control. Data are presented as mean ± SEM; n = 3 mice per group. *P < 0.05, ***P < 0.001, ****P < 0.0001, one-way ANOVA. (j) Representative immunofluorescence staining of CD31 and α-SMA in lung sections 14 days after bleomycin challenge, showing reduced CD31 and α-SMA co-expression in Adh5 EC-KO mice compared with control mice. Scale bar, 20 μm. (k) Masson's trichrome staining of lung sections showed reduced collagen deposition in *Adh5* EC-KO mice compared to littermate controls at 14 days post-BLM challenge. (l) Quantification of collagen area relative to total lung area in littermate control and *Adh5* EC-KO mice, both challenged with BLM. n ≥ 6 mice per group, mean ± SEM. *P < 0.05, Student's t-test. (m) Quantification of hydroxyproline levels in lung tissue using hydroxyproline assay kits, indicating reduced collagen content in *Adh5* EC-KO mice compared to littermate controls challenged with BLM. n ≥ 5 mice per group, mean ± SEM. *P < 0.05, Student's t-test.

To determine the role of endothelial GSNOR in fibrotic remodeling, we generated inducible endothelial-specific *Adh5* knockout (*Adh5* EC-KO) mice by crossing *Cdh5*-CreERT2 mice with *Adh5*^fl/fl^ mice, with *Adh*5^fl/fl^ littermates serving as controls (Fig. 4a, Fig. S7a and b). Following tamoxifen induction, lung ECs (CD45^−^CD31^+^CD144^+^) were isolated by flow cytometric sorting (FACS) to validate endothelial-specific deletion (Fig. 4b). *Adh5* mRNA and GSNOR protein levels were reduced to approximately 23% and 37% of control levels, respectively, confirming efficient endothelial deletion (Fig. 4c–e).

Following BLM challenge (2.5 U/kg, 14 days; Fig. 4f), control mice exhibited marked reductions in forced vital capacity (FVC) and chord compliance (Cchord), consistent with restrictive lung dysfunction. Endothelial *Adh5* deletion significantly attenuated the decline in FVC and partially preserved Cchord (Fig. 4g), indicating protection against BLM-induced pulmonary dysfunction.

Consistent with these functional improvements, *Adh5* EC-KO mice exhibited reduced expression of SNAI1 and α-SMA, and increased CD144 levels, compared with BLM-treated controls (Fig. 4h and i). Dual immunofluorescence further demonstrated reduced CD31/α-SMA co-localization in *Adh5* EC-KO lungs (Fig. 4j), supporting attenuation of EndoMT following endothelial GSNOR deletion.

Histological analysis using Masson's trichrome staining revealed a substantial reduction in collagen deposition in lung tissues of *Adh5* EC-KO mice compared to littermate controls (Fig. 4k and l). These results were further supported by hydroxyproline assays, which showed significantly lower collagen content in *Adh5* EC-KO lungs (Fig. 4m). BAL analysis of *Adh5* EC-KO mice exhibited a significant reduction in total leukocyte and neutrophil numbers, compared to controls at 14 days post-BLM treatment (Fig. S7c–d).

### 2.6 EC-specific overexpression of *Adh5* promotes EndoMT and pulmonary fibrosis

**Fig. 5:**
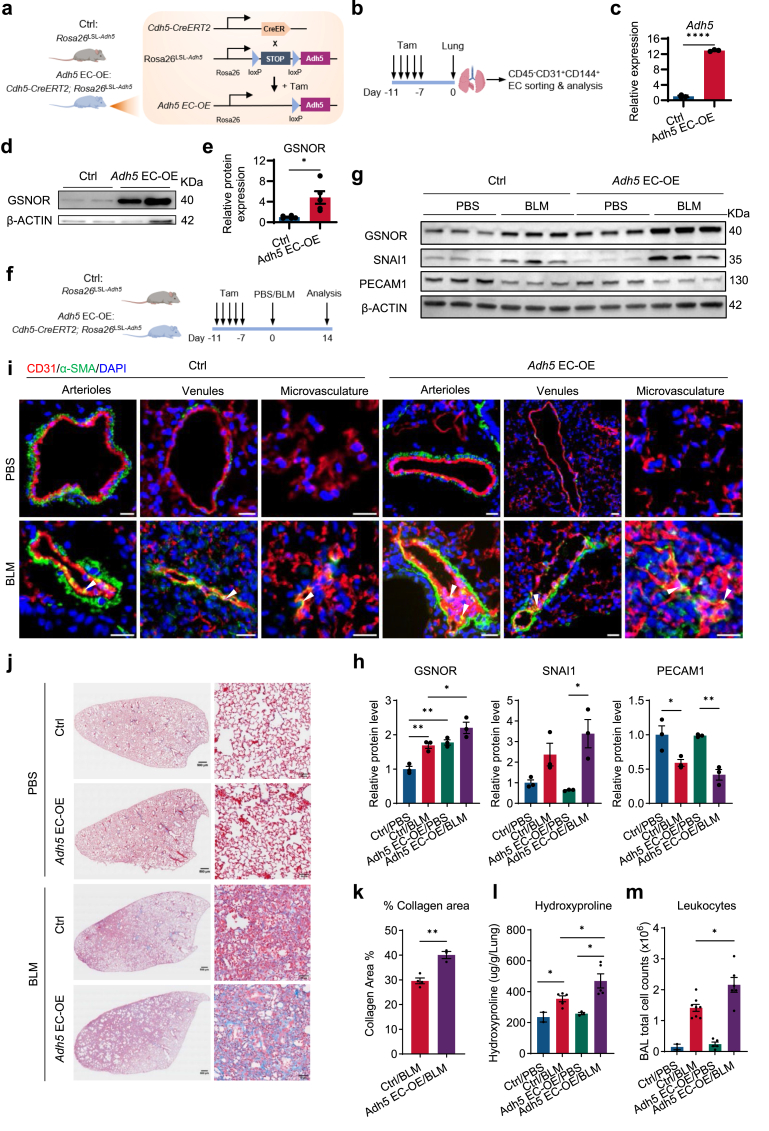
Endothelial Adh5 overexpression exacerbates EndoMT and bleomycin-induced pulmonary fibrosis. (a) Schematic representation of the CRISPR/Cas9 targeting strategy used to generate *Adh5* EC-specific overexpression (*Adh5* EC-OE) mice, in which Cre-mediated recombination removes the transcriptional STOP cassette upstream of the *Adh5* coding sequence at the *Rosa26* locus. *Adh5* EC-OE were defined as *Cdh5*-CreERT2; *Rosa26*^LSL−*Adh5*^ mice and *Rosa26*^LSL−*Adh5*^ littermate mice were used as controls. (b) Schematic of tamoxifen induction and lung EC sorting. Adh5 EC-OE mice and Rosa26^LSL−*Adh5*^ littermate controls received intraperitoneal injections of tamoxifen (100 mg/kg) for 5 consecutive days, followed by a 1-week waiting period to induce Adh5 overexpression in ECs. Lung ECs were sorted by flow cytometry as CD31^+^CD144^+^ cells. (c) qPCR analysis of Adh5 expression in sorted lung ECs from control and Adh5 EC-OE mice. Data are presented as mean ± SEM; n = 3 mice per group. ****P < 0.0001, Student's t-test. (d) Representative Western blot showing increased GSNOR protein levels in sorted lung ECs from Adh5 EC-OE mice compared with controls. n = 2 mice per group. (e) Densitometric quantification of GSNOR protein levels in sorted lung ECs. Protein levels were normalized to the corresponding loading control. Data are presented as mean ± SEM; n = 5 mice per group. *P < 0.05, Student's t-test. (f) Schematic of the bleomycin-induced lung fibrosis model. Mice were administered bleomycin intratracheally at 2.5 U/kg, and lung samples were collected 14 days post-challenge. (g) Western blot analysis showing increased SNAI1 expression and decreased PECAM1 expression in *Adh5* EC-OE mice compared to controls at 14 days post-BLM challenge. n = 3 mice per group. (h) Densitometric analysis of GSNOR, SNAIl, and PECAM1 protein levels. n = 3 mice per group, mean ± SEM. *P < 0.05, ***P < 0.001, ****P < 0.0001, one-way ANOVA. (i) Representative immunofluorescence staining of CD31 and α-SMA in lung sections 14 days after bleomycin challenge, showing increased CD31 and α-SMA co-expression in Adh5 EC-OE mice compared with control mice. Scale bar, 20 μm. (j) Masson's trichrome staining of lung sections showed increased collagen deposition in *Adh5* EC-OE mice compared to controls at 14 days post-BLM challenge. Scale bars, 500 μm (left) and 50 μm (right). (k) Quantification of collagen area relative to total lung area in littermate control and *Adh5* EC-OE mice, both challenged with BLM. n ≥ 3 mice per group, mean ± SEM. **P < 0.01, Student's t-test. (l) Quantification of lung hydroxyproline content showing increased collagen accumulation in Adh5 EC-OE mice compared with controls after BLM challenge. Data are presented as mean ± SEM; n ≥ 3 mice per group, except n = 2 for the PBS control group. *P < 0.05, one-way ANOVA. (m) Total leukocyte counts from 3 mL BAL fluid. n ≥ 5 mice per group, except n = 2 for the PBS control group, means ± SEM. *P < 0.05, one-way ANOVA.

To determine whether endothelial GSNOR upregulation is sufficient to promote fibrotic remodeling, we generated inducible endothelial-specific *Adh5* overexpression (*Adh5* EC-OE) mice by crossing *Cdh5*-CreERT2 mice with Rosa26^LSL−*Adh5*^ mice, with Rosa26^LSL−*Adh5*^ littermates serving as controls (Fig. 5a, Fig. S8a and b). Following tamoxifen induction, lung ECs (CD45^−^CD31^+^CD144^+^) were isolated by flow cytometric sorting to validate endothelial-specific overexpression (Fig. 5b). *Adh5* mRNA and GSNOR protein levels were increased by approximately 13-fold and 4.8-fold, respectively, confirming efficient endothelial overexpression (Fig. 5c–e).

Following BLM challenge, *Adh5* EC-OE mice exhibited increased SNAI1 expression and reduced CD144 levels, consistent with enhanced EndoMT (Fig. 5f–h). Dual immunofluorescence further demonstrated increased CD31/α-SMA co-localization in *Adh5* EC-OE lungs (Fig. 5i). Moreover, endothelial *Adh5* overexpression aggravated pulmonary fibrosis, as evidenced by increased collagen deposition and hydroxyproline content (Fig. 5j–l), and enhanced inflammatory responses, reflected by elevated BAL leukocyte counts (Fig. 5m and Fig. S8c and d).

### 2.7 Quantitative S-nitrosoproteomics reveals GSNOR-dependent remodeling in fibrosis

**Fig. 6:**
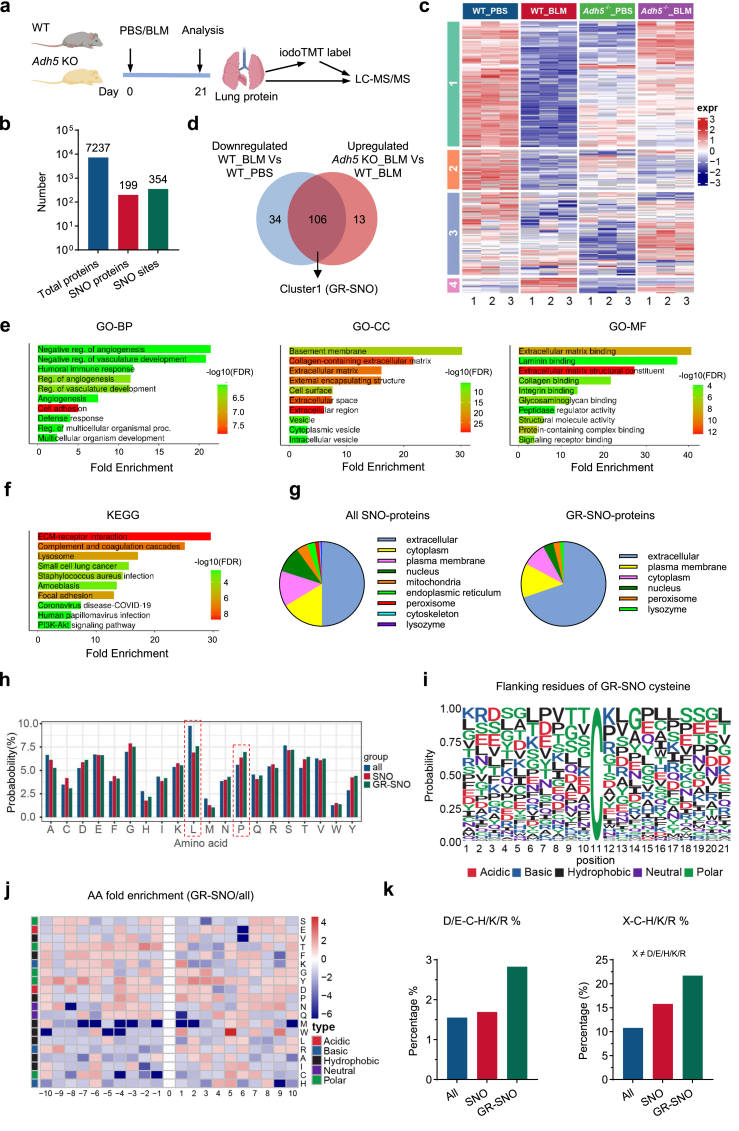
Protein nitrosylation is downregulated in BLM induced pulmonary fibrosis. (a) Schematic of the experimental design: WT littermate control and *Adh5* KO mice were challenged with 2.5 U/kg BLM or PBS intratracheally, and lung tissues were collected 21 days post-challenge. S-nitrosylated (SNO) proteins were analyzed using iodo-TMT labeling and followed by LC-MS/MS. n = 3 mice per group. (b) Bar plot illustrating the number of identified proteins, SNO-proteins, and SNO-sites across the experimental groups. (c) Heatmap showing four clusters of SNO sites: Cluster 1 (downregulated in WT_BLM vs. WT_PBS and upregulated in *Adh5* KO_BLM vs. WT_BLM); Cluster 2 (downregulated in WT_BLM vs. WT_PBS and either unchanged or further downregulated in *Adh5* KO_BLM vs. WT_BLM); Cluster 3 (unchanged in WT_BLM vs. WT_PBS); and Cluster 4 (upregulated in WT_BLM vs. WT_PBS). n = 3 mice per group. (d) Venn diagram showing 106 overlapping SNO sites (Cluster 1) that were downregulated in WT_BLM vs. WT_PBS and upregulated in *Adh5* KO_BLM vs. WT_BLM, referred to as GSNOR-regulated SNO sites (GR-SNO sites). (e) GO enrichment analysis of proteins from Cluster 1, which contain GR-SNO sites and are thus defined as GR-SNO proteins. (f) KEGG pathway enrichment analysis of the same set of GR-SNO proteins described in (e). (g) Subcellular distribution of all SNO proteins and GR-SNO proteins in lung tissues. (h) Amino acid composition in whole lung proteins and in the ±10 amino acid sequences flanking validated S-nitrosylated cysteine sites in SNO and GR-SNO proteins. Whole lung protein composition represents the overall percentage of each amino acid, whereas SNO and GR-SNO compositions reflect the amino acid distribution specifically within the sequences surrounding experimentally verified S-nitrosylated cysteines. (i) Sequence logo showing the amino acid composition flanking GR-SNO sites (±10 positions surrounding validated S-nitrosylated cysteines). Letter size represents the relative frequency of each amino acid at the corresponding position. (j) Amino acid fold enrichment at each position flanking GR-SNO cysteines. Fold enrichment was calculated as the ratio of amino acid composition at each of the ±10 positions surrounding GR-SNO cysteines to the overall amino acid composition in whole lung proteins. (k) Percentage of D/E-C-H/K/R and X-C-H/K/R motifs in whole lung proteins, SNO proteins, and GR-SNO proteins. Here, X represents a non-charged amino acid. In whole lung proteins, the percentage reflects the occurrence of these motifs across all proteins, whereas in SNO and GR-SNO proteins, the percentage is based only on sequences flanking experimentally validated S-nitrosylation sites.

To determine whether GSNOR regulates EndoMT via protein denitrosylation, we analyzed S-nitrosylated protein profiles in fibrotic lung tissues collected 21 days post-BLM and compared them to PBS-treated control lung tissues in both *Adh5* KO mice and WT littermate controls. S-nitrosylated cysteine sites were detected using an iodo-TMT-based S-nitrosylation switch approach followed by LC–MS/MS analysis [18] (Fig. 6a), while total protein abundance was measured independently by TMT-based proteomics. After normalization of site-level S-nitrosylation to the corresponding protein abundance, we identified 199 SNO-proteins across 7237 total proteins, encompassing 354 SNO sites (Fig. 6b).

In WT BLM-treated lung tissues, the SNO levels at 140 cysteine sites were significantly downregulated compared with the WT-PBS group, consistent with increased GSNOR activity and denitrosylation events. In *Adh5* KO BLM-treated lungs, the SNO levels in 119 cysteine sites were upregulated compared to WT BLM-treated lungs. Notably, 106 cysteine sites overlapped between the 140 and 119 sites (Fig. 6c and d). These overlapping sites were defined as GSNOR-regulated S-nitrosylation sites (GR-SNO sites; cluster 1, 76 proteins), showing decreased S-nitrosylation during fibrosis that was restored or increased upon *Adh5* deletion (Fig. 6c and d).

Importantly, the 76 proteins containing GR-SNO sites, hereafter referred to as GR-SNO proteins, were strongly associated with endothelial function and extracellular matrix (ECM) remodeling, as shown by GO and KEGG pathway enrichment in endothelial functions (e.g., vasculature development, cell adhesion, and angiogenesis), and in fibrosis-related pathways (e.g., ECM-receptor interaction and ECM binding) (Fig. 6e and f), linking GSNOR-dependent S-nitrosylation to endothelial dysfunction and fibrotic ECM remodeling.

Subcellular distribution analysis revealed that GR-SNO proteins were predominantly located in extracellular compartments, followed by the cytoplasm, plasma membrane, nucleus, mitochondria, ER, peroxisome, cytoskeleton, and lysosome. This may reflect cellular NO or GSNO availability. Interestingly, GR-SNO proteins exhibited a similar distribution pattern, except that mitochondrial and ER proteins were less represented in this subset. This aligns with prior reports that mitochondrial denitrosylation is primarily mediated by the thioredoxin reductase system [19] rather than GSNO (Fig. 6g).

To further elucidate the molecular determinants of SNO- and GR-SNO-protein susceptibility, we analyzed the amino acid composition of full-length proteins (Fig. S9a) and the 20 amino acids (Fig. 6h–j and Fig. S9b–d) flanking the SNO or GR-SNO sites (10 AA upstream and 10 AA downstream). Comparative analysis revealed proline (P) enrichment in SNO and GR-SNO proteins, while leucine (L) was depleted relative to total lung proteins (Fig. 6h and i). Interestingly, GR-SNO sites exhibited even higher enrichment of P, suggesting this feature may facilitate active SNO switching. Indeed, the proline structure is known to aid protein folding by creating rigid turns in the peptide chain and defining the boundaries of β-sheets and α-helices [20]. In contrast, reduced leucine content near modified cysteine residues (Fig. 6i) may reflect a local sequence environment associated with S-nitrosylation susceptibility.

Sequence motif analysis revealed a preference for the **X-C-(K/R/H)** motif, where a non-charged residue precedes the cysteine and a basic residue follows. This motif was highly enriched, occurring in 15.8% of SNO proteins and 21.7% of GR-SNO proteins (Fig. 6k and Fig. S9e–f). This enrichment suggests that basic residues adjacent to cysteines may enhance trans-nitrosylation with GSNO by promoting electrostatic interactions. These findings align with previous studies indicating that charged residues facilitate S-nitrosylation events [[21], [22], [23]].

### 2.8 GSNOR reduces high mobility group box 1 (HMGB1) S-nitrosylation at C23 during EndoMT

**Fig. 7:**
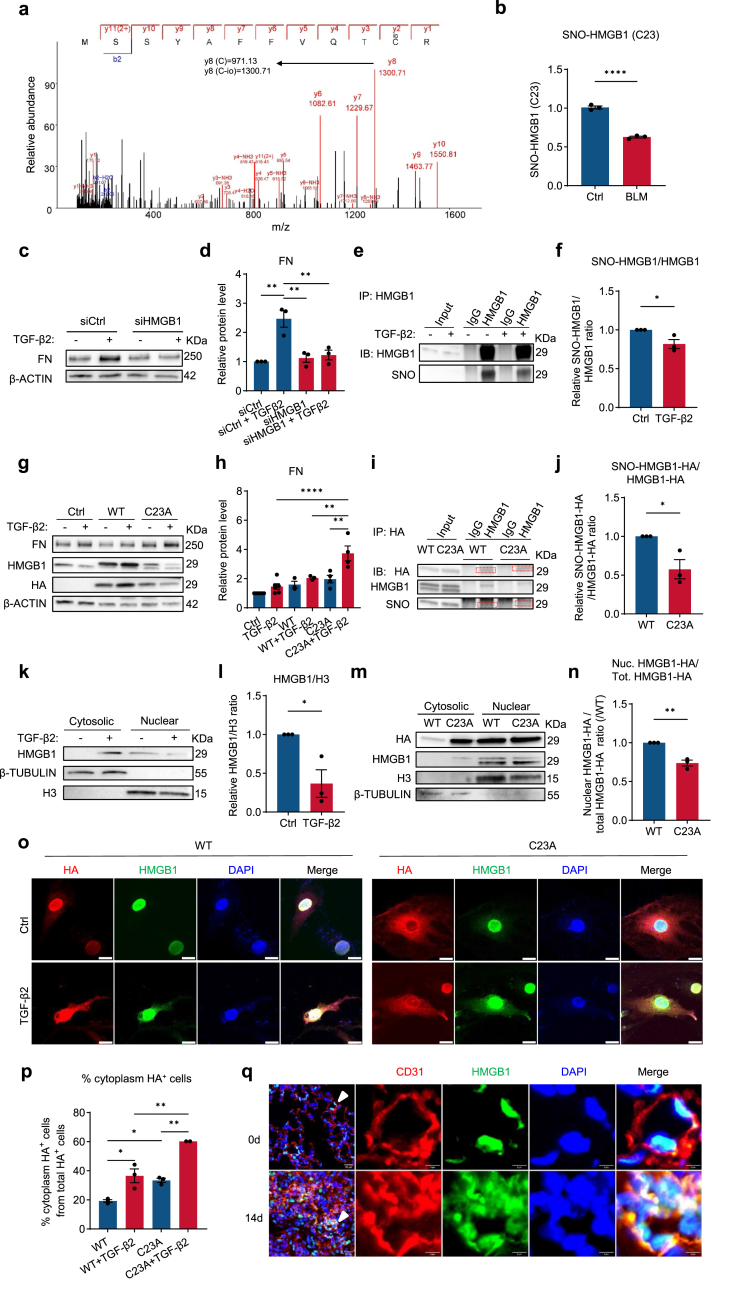
HMGB1 S-nitrosylation mediates EndoMT. (a) Identification of nitrosylated HMGB1 at Cysteine-23 (C23) in murine lung tissues by iodo-TMT labeling and LC-MS/MS. (b) Relative SNO-HMGB1(C23) level in lung tissues of control and BLM-induced mice after 21 days. n = 3 mice per group, mean ± SEM. ****P < 0.0001, Student's t-test. (c) Western blot analysis showing decreased FN protein levels in HPMECs with HMGB1 knockdown for 48 h, followed by 24-h stimulation with 10 ng/mL TGF-β2. (d) Densitometric analysis of FN protein levels. n = 3 independent biological replicates per group, mean ± SEM. **P < 0.01, one-way ANOVA. (e) Immunoblot showing reduced nitrosylation of HMGB1 upon 24-h 10 ng/mL TGF-β2 stimulation in HPMECs, as assessed by immunoprecipitation with an anti-HMGB1 antibody and immunoblotting with anti-HMGB1 and anti-SNO antibodies. (f) Densitometric analysis of SNO-HMGB1 protein levels in HPMECs. The ratio of SNO-HMGB1 to total HMGB1 was quantified, normalized to the control group, and showed a reduction upon TGF-β2 stimulation. n = 3 independent biological replicates per group, mean ± SEM. *P < 0.05, Student's t-test. (g) Overexpression of HA-tagged HMGB1(WT) and HMGB1(C23A) for 48 h via lentivirus in HPMECs, followed by 10 ng/mL TGF-β2 stimulation for 6 h. Western blot analysis showing increased FN levels in HMGB1(WT)- and HMGB1(C23A)-overexpressing cells post-TGF-β2 stimulation, with FN levels higher in HMGB1(C23A)-overexpressing cells. (h) Densitometric analysis of FN protein levels. FN expression was significantly higher in HMGB1(C23A)-overexpressing cells than in HMGB1(WT)-overexpressing cells under TGF-β2 stimulation. n ≥ 3 independent biological replicates per group, mean ± SEM. **P < 0.01, ****P < 0.0001, one-way ANOVA. (i) Immunoprecipitation with an anti-HA antibody followed by Western blot analysis using anti-HA, anti-HMGB1, and anti-SNO antibodies showing reduced SNO-HMGB1-HA levels in HMGB1(C23A)-overexpressing cells compared to HMGB1(WT)-overexpressing cells. (j) Densitometric analysis of SNO-HMGB1-HA protein levels. The ratio of nitrosylated HMGB1-HA to total HMGB1-HA was quantified, with the HA band representing total HMGB1-HA levels. n = 3 independent biological replicates per group, mean ± SEM. *P < 0.05, Student's t-test. (k) Immunoblot analysis of nuclear and cytoplasmic fractions showing HMGB1 nuclear export in HPMECs after 24-h stimulation with 10 ng/mL TGF-β2. (l) Densitometric analysis of nuclear HMGB1 levels. Nuclear HMGB1 was first normalized to H3 and then compared between the control and TGF-β2-treated groups. A significant decrease was observed after TGF-β2 stimulation. n = 3 independent biological replicates per group, means ± SEM. *P < 0.05, Student's t-test. (m) Immunoblot showing that overexpression of HMGB1(C23A)-HA resulted in a lower proportion of nuclear HMGB1-HA compared to HMGB1(WT)-HA in HPMECs. (n) Densitometric quantification of the ratio of nuclear HMGB1-HA (Nuc. HMGB1-HA) to total HMGB1-HA (Tot. HMGB1-HA, including both nuclear and cytoplasmic fractions), showing a significant decrease in HMGB1(C23A)-overexpressing cells compared to HMGB1(WT)-overexpressing cells. n = 3 independent biological replicates per group, means ± SEM. **P < 0.01, Student's t-test. (o) Representative immunofluorescence analysis showing the distribution of HMGB1(WT)-HA and HMGB1(C23A)-HA in overexpressing HPMECs with or without 10 ng/mL TGF-β2 stimulation. Scale bar = 10 μm. (p) Quantification of the ratio of cytoplasmic HA-positive cells (indicating cytoplasmic localization of overexpressed HMGB1) to total HA-positive cells (indicating all successfully transduced cells, regardless of localization). n = 3 independent biological replicates per group, except n = 2 for the HMGB1(C23A) + TGF-β2 group. Mean ± SEM. *P < 0.05, **P < 0.01, one-way ANOVA. (q) WT mice were administered 5 U/kg BLM intratracheally. Lung tissues were collected 14 days post-challenge. Immunofluorescence staining of formalin-fixed, paraffin-embedded lung sections with anti-CD31, anti-HMGB1, and DAPI revealed increased HMGB1 translocation to the cytoplasm in ECs. Bar = 5 μm.

To explore whether GSNOR mediates EndoMT through the denitrosylation of a key protein, we focused on HMGB1 (Fig. 7a and b), a nuclear protein previously implicated in promoting EndoMT and pulmonary fibrosis [24,25]. Using our *in vitro* EndoMT model, we confirmed that TGF-β2 stimulation of HPMECs upregulated HMGB1 expression, contributing to EndoMT (Fig. S3a–e). Conversely, *HMGB1* knockdown by siRNA (Fig. S10a–b) inhibited FN upregulation (Fig. 7c and d), highlighting HMGB1's essential role in this process.

Interestingly, we found that HMGB1's SNO levels were significantly downregulated during EndoMT *in vitro* and fibrosis development *in vivo*. In quiescent lung tissue, HMGB1 was predominantly S-nitrosylated at C23, a conserved site across species, including humans and mice (Fig. S10c), while S-nitrosylation at Cysteine-45 (C45) or Cysteine-106 (C106) was not confidently detected in this dataset. Following BLM administration, HMGB1 S-nitrosylation at C23 was markedly reduced (Fig. 7a and b). Similarly, *in vitro*, TGF-β2-stimulated HPMECs showed a significant decrease in the proportion of nitrosylated HMGB1 (Fig. 7e and f), indicating that HMGB1 denitrosylation contributes to EndoMT.

Based on these findings, we hypothesized that GSNOR-associated reduction of HMGB1 C23 S-nitrosylation promotes EndoMT. To test this hypothesis, we constructed HA-tagged HMGB1 plasmids—wild-type (HMGB1-WT) and a C23A mutant (HMGB1-C23A), in which C23 was mutated to alanine and generated lentiviruses for efficient delivery (Fig. S10d–e). Overexpression of WT and C23A *HMGB1* in HPMECs led to a robust 20-fold increase in *HMGB1* expression (Fig. S10f–g) and significantly elevated FN levels upon TGF-β2 stimulation. Notably, the FN level was markedly higher in C23A-overexpressing cells than WT cells (Fig. 7g and h), suggesting that the loss of nitrosylation at C23 enhances HMGB1's pro-EndoMT activity. For further validation, immunoprecipitation studies confirmed that overexpressed HMGB1-C23A proteins exhibited reduced SNO levels compared with HMGB1-WT proteins (Fig. 7i and j). These findings support the interpretation that C23 nitrosylation of HMGB1 inhibits, whereas loss of C23 S-nitrosylation promotes EndoMT.

### 2.9 Loss of HMGB1 C23 S-nitrosylation promotes HMGB1 translocation and EndoMT

To investigate the distribution of HMGB1 during EndoMT, we first examined its nuclear and cytosolic localization following TGF-β2 stimulation. Western blot analysis revealed HMGB1 translocation from the nucleus to the cytoplasm upon TGF-β2 stimulation (Fig. 7k and l). When HMGB1-WT or HMGB1-C23A was overexpressed using lentivirus, HMGB1-WT predominantly localized to the nucleus, whereas a significant proportion of HMGB1-C23A was detected in the cytoplasm. Endogenous HMGB1, meanwhile, remained primarily nuclear (Fig. 7m and n).

Immunofluorescence analysis further supported these observations. In the absence of TGF-β2 stimulation, cytoplasmic HMGB1 was observed in 19% of HMGB1-WT-overexpressing cells, compared to 33% in HMGB1-C23A-overexpressing cells (Fig. 7o and p). Upon TGF-β2 stimulation, these proportions increased to 37% in HMGB1-WT-overexpressing cells and 60% in HMGB1-C23A-overexpressing cells (Fig. 7o and p). Together, these findings indicate that denitrosylation of HMGB1 at C23 significantly enhances its nuclear-to-cytoplasmic translocation.

*In vivo* analysis of lung sections from BLM-challenged mice further corroborated these results. Cytoplasmic localization of HMGB1 was increased in microvasculature ECs (Fig. 7q), whereas HMGB1 predominantly localized to the nucleus in quiescent lung tissues.

## 3 Discussion

In this study, we show that GSNOR is aberrantly upregulated in ECs in a murine bleomycin-induced lung fibrosis model and in human IPF patient samples. Using inducible, EC-specific GSNOR gain- and loss-of-function transgenic mice, and pharmacologic GSNOR inhibitors, we demonstrate that GSNOR promotes EndoMT and exacerbates fibrotic remodeling *in vivo*. This upregulation is accompanied by local and systemic GSNO depletion and widespread remodeling of protein S-nitrosylation.

Specifically, reduced S-nitrosylation of the key damage-associated molecular pattern (DAMP) molecule HMGB1 at C23 facilitates its nuclear-to-cytosol shuttle and amplifies TGF-β–driven EndoMT. Under basal conditions, SNO-HMGB1 at C23 may help maintain nuclear localization, whereas loss of this modification promotes its cytosolic redistribution, thereby amplifying TGF-β–driven EndoMT. Although multiple post-translational modifications are known to influence HMGB1 trafficking [26,27], our findings reveal S-nitrosylation as a previously unrecognized, site-specific regulatory mechanism that controls its compartmentalization and signaling in ECs.

GSNOR upregulation has been reported in multiple pulmonary diseases [[28], [29], [30], [31]], including asthma and COPD, and pharmacological inhibition shows protective effects [6,[31], [32], [33], [34]], underscoring its critical role in maintaining pulmonary homeostasis. Consistently, analysis of our own and independent IPF patient cohorts [16], together with bleomycin-induced murine lung fibrosis models, revealed markedly elevated GSNOR expression in microvascular ECs, closely associated with EndoMT. This upregulation coincides with local and systemic depletion of GSNO and widespread remodeling of protein S-nitrosylation, involving 106 GR-SNO sites across 76 proteins.

Sequence motif analysis further revealed local amino acid features associated with GR-SNO sites, suggesting sequence-dependent susceptibility to dynamic S-nitrosylation regulation, whereas mitochondrial and endoplasmic reticulum proteins were less represented in this subset, suggesting compartment-selective SNO remodeling. The observed increase in GSNOR likely represents an adaptive response to excessive reactive nitrogen species (RNS) and oxidative stress in IPF [35,36]. However, this compensatory mechanism appears maladaptive, as it is associated with altered S-nitrosylation of proteins involved in endothelial stability [37], thereby promoting endothelial fate switching and fibrotic remodeling.

Under physiological conditions, the lung S-nitrosoproteome comprises multiple intracellular DAMP-related proteins, such as HMGB1, S100 proteins, histones, and heat shock proteins. DAMPs are usually sequestered by compartmentalization and can be released or redistributed upon cellular stress or injury [38,39]. Although multiple signaling pathways regulate DAMP localization and release [[40], [41], [42]], whether redox-dependent S-nitrosylation contributes to this process during fibrotic endothelial remodeling remains incompletely understood.

Our study suggests that GSNOR-associated S-nitrosylation remodeling may regulate this process, with HMGB1 serving as one experimentally supported downstream effector. Increased endothelial GSNOR was associated with reduced HMGB1 S-nitrosylation and enhanced HMGB1 cytoplasmic redistribution. Functional modulation of HMGB1 affected EndoMT-associated marker expression, supporting HMGB1 as a key downstream effector of GSNOR-associated endothelial remodeling. However, whether other S-nitrosylated DAMP-related proteins identified by proteomics are directly regulated by GSNOR or contribute functionally to EndoMT remains to be determined.

GSNOR functions as a regulator of the endothelial S-nitrosoproteome, influencing the extent and localization of protein S-nitrosylation. By regulating GSNO metabolism, low GSNOR levels help maintain S-nitrosylation homeostasis. In fibrotic settings, GSNOR upregulation disrupts this equilibrium, shifting toward reduced protein S-nitrosylation and destabilizing endothelial identity. Reduced S-nitrosylation of nuclear and cytosolic proteins such as HMGB1 may promote EndoMT-associated endothelial remodeling.

Several limitations should be acknowledged. First, fibrosis outcomes were assessed at selected time points after bleomycin challenge; longitudinal studies are needed to define the kinetics of endothelial GSNOR in fibrosis development and resolution. Second, although our data support EndoMT-associated endothelial remodeling, the contribution of endothelial-derived α-SMA^+^ myofibroblasts was not quantified, leaving the extent of EndoMT unresolved. Finally, although the bleomycin model does not fully recapitulate chronic human IPF, increased endothelial ADH5/GSNOR expression in IPF lungs supports clinical relevance and warrants validation in chronic and therapeutic models.

In conclusion, we identify GSNOR as a key regulator of endothelial S-nitrosylation remodeling and endothelial fate. Aberrant GSNOR upregulation shifts protein S-nitrosylation from a homeostatic, regenerative axis toward a profibrotic, fate-altering program. These findings support further investigation of GSNOR-regulated S-nitrosothiol metabolism as a potential strategy to modulate endothelial remodeling in fibrotic lung disease.

## 4 Materials and methods

### 4.1 Human lung tissues

All human samples were obtained in accordance with the principles of the Declaration of Helsinki. The study protocol was approved by the Ethics Committee of the First Affiliated Hospital of Guangzhou Medical University. Specimens from IPF patients were collected during lung transplantation surgeries or biopsies, while healthy lung tissues were acquired from organ donors diagnosed with brain death. In all cases, written consent for tissue use was obtained from the patients or their family members.

### 4.2 Mice

All mouse experiments were approved by the Institutional Animal Care and Use Committee of the Guangzhou National Laboratory and were conducted in accordance with Directive 2010/63/EU of the European Parliament on the protection of animals used for scientific purposes. All mice were kept under specific pathogen-free conditions in the central animal facility of the Guangzhou National Laboratory. Male mice between 10 and 14 weeks of age were used in all *in vivo* experiments to minimize potential sex-related variability in tamoxifen-induced Cre recombination efficiency and downstream experimental outcomes, as has been reported in inducible Cre models. *Cdh5-*creERT2 [43], R26R-tdTomato [44] mice were previously described.

*Adh5* KO mice were generated using CRISPR/Cas9 technology to knock out exon 3 of the *Adh5* gene and introduce a frameshift mutation through non-homologous end joining (NHEJ). The Cas9 mRNA and sgRNA were transcribed *in vitro* and microinjected into fertilized eggs of C57BL/6J mice to produce F0 mice. sgRNA sequences are listed in Table 1. Positive F0 mice were identified through PCR amplification and Sanger sequencing and subsequently bred with C57BL/6J mice to obtain F1 offspring. *Adh5*^*+/−*^ heterozygous F1 mice were backcrossed to C57BL/6J mice for six generations, and the colony was then maintained by intercrossing *Adh5*^*+/−*^ heterozygotes.

*Adh5*^fl/fl^ mice were generated using homologous recombination to flox the *Adh5* gene by inserting loxP sites flanking exon 3. Cas9 mRNA and gRNA were synthesized *in vitro*, and a donor vector was constructed using In-Fusion cloning. The donor vector contained a 2.9 kb 5′ homology arm, a 0.8 kb floxed region, and a 3.0 kb 3′ homology arm. The Cas9 mRNA, sgRNA, and donor vector were microinjected into C57BL/6J mouse zygotes. sgRNA sequences are listed in Table 1. F0 mice were screened by PCR and sequencing to identify successful edits. Positive F0 mice were bred with C57BL/6J mice to obtain F1 offspring. *Adh5*^fl/-^ heterozygous F1 mice were backcrossed to C57BL/6J mice for six generations, and the colony was then maintained by intercrossing *Adh5*^fl/-^ heterozygotes.

*Rosa26* ^LSL−*Adh5*^ mice were generated using CRISPR/Cas9 technology to insert the CAG-LSL-*Adh5*-WPRE-pA expression cassette into the *Rosa26* locus via homologous recombination. Cas9 mRNA and sgRNA were synthesized *in vitro*, and a donor vector containing a 3.3 kb 5′ homology arm, the expression cassette, and a 3.3 kb 3′ homology arm was constructed using In-Fusion cloning. The Cas9 mRNA, gRNA, and donor vector were microinjected into C57BL/6J mouse zygotes to generate F0 mice. sgRNA sequences are listed in Table 1. Positive F0 mice were identified through PCR and Sanger sequencing and bred with C57BL/6J mice to produce F1 offspring. *Rosa26* ^LSL*-Adh5/+*^ heterozygous F1 mice were backcrossed to C57BL/6J mice for six generations, and the colony was then maintained by intercrossing *Rosa26* ^*LSL-Adh5/+*^ heterozygotes. All new genetic mice were generated by Shanghai Model Organisms Center, Inc.

To induce recombination, tamoxifen (Sigma-Aldrich, USA) at a dose of approximately 100 mg/kg body weight was administered via intraperitoneal injection once every 24 h for five consecutive days. Seven days after the final tamoxifen injection, these mice were subjected to experiments. All experimental and littermate control mice received the same tamoxifen regimen to control for potential tamoxifen-related effects across genotypes. *Cdh5*-creERT2; *R26R*-tdTomato mice were used for EC lineage tracing. *Adh5* EC-KO (*Cdh5*-creERT2; *Adh5*^fl/fl^) mice were used for EC-specific knockout of *Adh5*, and *Adh5* EC-OE (*Cdh5*-creERT2; *Rosa26* ^LSL−*Adh5*^) mice were used for EC-specific overexpression of *Adh5*.

### 4.3 Anesthesia and euthanasia

Mice were anesthetized with pentobarbital sodium (50 mg/kg, intraperitoneal injection) prior to experimental procedures. At experimental endpoints, animals were euthanized by overdose of pentobarbital sodium (200 mg/kg, intraperitoneal injection), followed by carbon dioxide inhalation using a gradual-fill method (20–30% of the chamber volume per minute), resulting in loss of consciousness within 2–3 min. Death was confirmed by the absence of cardiac activity and reflexes.

### 4.4 Cells

HPMECs (FHHUM104, Fenghui Biotechnology Co., Ltd., China) were cultured in EGM™-MV BulletKit™ Medium (CC-3125, Lonza, Switzerland) following the manufacturer's instructions. Cells from passages 5 to 10 were used in all experiments. HEK293T cells were obtained from the American Type Culture Collection (ATCC, USA; CRL-3216) and were routinely tested for mycoplasma contamination.

### 4.5 *In vitro* EndoMT assay

HPMECs were maintained in EGM™-MV BulletKit™ Medium at 37°C with 5% CO2. The cells were cultured until they reached approximately 70% confluency and then treated with or without TGF-β2 (10 ng/mL, Cat# 302-B2-010/CF, R&D Systems) for 6 h, 24 h, or 72 h. After treatment, cells were collected and analyzed for the expression of endothelial and mesenchymal markers by qPCR and Western blot. In some experiments, 1 μM N6022 was added to the cells and incubated for 1 h prior to TGF-β2 treatment.

### 4.6 siRNA-mediated gene silencing

When HPMECs reached approximately 70% confluency, 300 pmol of siRNA was transfected using Lipofectamine RNAiMAX (Cat# 13778150, Invitrogen) in EBM-2 Basal medium (Cat# CC-3156, Lonza) for 6 h. Following the incubation, the medium was replaced with EGM™-MV BulletKit Medium, and the cells were cultured for an additional 48 h. Three different siRNA duplexes were designed, and mixed siRNAs were used for each targeted gene. The siRNA sequences are listed in Table 2.

### 4.7 BLM-induced pulmonary fibrosis model

A 50 μL BLM solution (5 U/kg; Selleck Chemicals, USA) was administered intratracheally after mice were anesthetized. Control mice received PBS under the same conditions. For genetically modified mice (*Adh5* KO, *Adh5* EC-KO, *Adh5* EC-OE), a reduced BLM dose of 2.5 U/kg was used. In some experiments, mice were pretreated with the GSNOR inhibitor N6022 ^31^ (1 mg/kg or 30 mg/kg; Cat# B1111, APExBIO, USA) via intraperitoneal injection one day before the BLM challenge. Alternatively, mice were pretreated with the GSNOR inhibitor N91115 ^33^ (Cavosonstat; 1 mg/kg, 5 mg/kg, or 10 mg/kg; Cat# DC11518, DC Chemicals, China) orally one day before a 5 U/kg BLM challenge, followed by oral administration of N91115 every two days post-challenge.

### 4.8 Pulmonary function measurements

The trachea was surgically exposed in anesthetized mice, and a cannula was inserted and secured with a suture to prevent air leakage. Mice were placed in the chamber of the Buxco FinePointe RC system (Data Sciences International, St. Paul, MN, USA) and connected to a ventilator. Mechanical ventilation was initiated at a respiratory rate of approximately 135 breaths/min. Cchord and FVC were recorded according to the manufacturer's protocol.

### 4.9 BAL

BAL samples were obtained by three sequential 1 mL lavages with ice cold PBS with 2 mM EDTA and 2% FBS. The 1st mL of BAL supernatant was frozen at −80^°^C for ELISA. After red cell lysis with red cell lysis buffer (Cat# RT122, TIANGEN, China), pooled cells from all 3 mL were used to enumerate cell counts by hemocytometer.

### 4.10 GSNO ELISA

Whole lungs were collected post-lavage, snap-frozen in liquid nitrogen, homogenized with steel beads in 1 mL PBS, and centrifuged at 12,000 rpm for 20 min at 4°C. The supernatant was stored at −80°C. GSNO levels in BAL fluid, lung homogenate supernatant, and plasma were measured using the Mouse GSNO ELISA Kit (Cat# MM-45702M1, MEIMIAN, China).

### 4.11 Vector construction

The pLV-CMV.EGFP.PGK.Puro plasmid (Cat# 13538) was obtained from PackGene Biotechnology (China). DNA sequences encoding GSG-P2A-hHMGB1-HA and GSG-P2A-hHMGB1(C23A, ACA→CGC)-HA, synthesized with *Bsr*GI and *Xba*I restriction sites and lacking stop codons in the *HMGB1* sequences, were inserted into the pLV-CMV.EGFP.PGK.Puro vector using these sites. This resulted in the construction of the pLV-CMV.EGFP-P2A-hHMGB1-HA.PGK.Puro and pLV-CMV.EGFP-P2A-hHMGB1(C23A)-HA.PGK.Puro plasmids.

### 4.12 Lentiviral production and transduction

Lentiviruses were produced by transfecting HEK293T cells with the following plasmids: 6 μg of an expression plasmid (either pLV-CMV.EGFP.PGK.Puro, pLV-CMV.EGFP-P2A-hHMGB1-HA.PGK.Puro, or pLV-CMV.EGFP-P2A-hHMGB1(C23A)-HA.PGK.Puro), 4.5 μg of psPAX2 (Addgene Cat# 12260), and 1.5 μg of pMD2.G (Addgene Cat# 12259), both of which were gifts from Didier Trono. HEK293T cells were cultured in DMEM (Gibco, Cat# C11965500CP) supplemented with high glucose, l-glutamine, and 10% FBS. When the cells reached approximately 70% confluency, they were transfected with the plasmid mixture using Lipofectamine 2000 (Invitrogen, Cat# 11668019) in basal DMEM for 6 h. After transfection, the medium was replaced with complete DMEM, and the cells were incubated for an additional 48 h. The supernatant containing lentiviral particles was then collected, filtered through a 0.45 μm PES filter, and concentrated using a Lentivirus Concentration Reagent (Yeason, Cat# 41101ES50) according to the manufacturer's instructions. The concentrated lentiviral stocks were stored at −80°C. HPMECs were transduced with the lentivirus 48 h post-production. Transduction efficiency was visually confirmed by the EGFP expression, with an efficacy of approximately 90%. The expression of hHMGB1-HA or hHMGB1(C23A)-HA was further validated by qPCR and immunoblot analysis.

### 4.13 Flow cytometry and cell sorting

BAL or lung single-cell suspensions were incubated with purified Rat anti-mouse CD16/CD32 (Mouse BD Fc Block, clone 2.4G2, BD Pharmingen) to block Fc-receptors, and stained as outlined below: For immune cell analysis, the following antibodies were used: BV421 Rat anti-mouse CD45 antibody (clone 30-F11, BD Horizon), BV510 Rat anti-mouse Ly6G antibody (clone 1A8, BD Horizon), PECy7 Hamster anti-mouse CD11c antibody (clone HL3, BD Pharmingen), APC Rat anti-mouse CD11b antibody (clone M1/70, eBioscience), PE Rat anti-mouse Siglec-F antibody (clone E50- 2440, BD Pharmingen), PerCP/Cy5.5 Rat anti-mouse Ly-6C antibody (clone HK1.4, Biolegend), APC-Cy7 Hamster Anti-Mouse CD3e (Clone 145-2C11, BD Pharmingen), FITC anti-mouse/human CD45R/B220 Antibody (Clone RA3-6B2, Biolegend). T cells were identified as CD45^+^CD11b^−^CD3e^+^, while B cells were identified as CD45^+^CD11b^−^B220^+^. Eosinophils and AMs were identified as CD45^+^CD11c^−^SiglecF^+^ and CD45^+^CD11c^+^SiglecF^+^, respectively. Proinflammatory monocytes were identified as CD45^+^CD11b^+^SiglecF^−^Ly6C^high^Ly6G^−^, and neutrophils were identified as CD45^+^CD11b^+^SiglecF^−^Ly6C^int^Ly6G^+^. For ECs sorting from lung tissue, a separate staining panel was used, including BV421 Rat anti-mouse CD45 antibody (clone 30-F11, BD Horizon), APC Rat anti-mouse CD31 antibody (clone MEC 13.3, BD Pharmingen), and PE Rat anti-mouse CD144 antibody (clone 11D4.1, BD Pharmingen). ECs were identified and sorted as CD45^−^CD31^+^CD144^+^ cells. Intracellular staining for α-SMA (Mouse Monoclonal Anti-α-SMA, Sigma A2547; Donkey Anti-Mouse IgG AF-488, Abcam 150105; Mouse control IgG, Abclonal, AC011) was conducted following previously established protocols [45]. Data were collected on CytoFLEX S Flow Cytometer (Beckman Coulter, USA) or BD LSRFortessa™ X-20 Cell Analyzer Flow Cytometry (BD) and analyzed by FlowJo (Tree Star Inc.). In some experiments, tdTomato^+^ cells or CD45^−^CD31^+^CD144^+^ ECs were sorted using a BD FACSAria III cell sorter (BD).

### 4.14 Real-time PCR

Human lung tissue RNA was extracted using the AllPrep DNA/RNA/Protein Mini Kit (Cat# 80004, QIAGEN). For mouse lung tissue and HPMEC RNA, extraction was performed with the FastPure Cell/Tissue Total RNA Isolation Kit V2 (Cat# RC112-01, Vazyme, China). cDNA was synthesized using the HiScript III RT SuperMix for qPCR (+gDNA wiper) kit (Cat# R323-01, Vazyme, China). Quantitative PCR (qPCR) was carried out with SYBR® Green Real-Time PCR Master Mix (Cat# QPK-201, Toyobo, Japan) on a QuantStudio™ 3 Real-Time PCR system (Applied Biosystems, USA). The primers used are listed in Table 3. The expression of target genes was normalized using 18S rRNA as an internal control.

### 4.15 Immunoprecipitation

Mice lung tissues were homogenized in 1 mL of Pierce® IP Lysis Buffer (Cat# 87787, Thermo Fisher Scientific, USA) with steel beads, supplemented with EDTA-Free Protease Inhibitor Cocktail (Cat# B14002, Selleck, USA), and centrifuged at 14,000g for 20 min at 4°C to remove cell debris. The lysates were precleared by adding 100 μL of equilibrated Dynabeads Protein G beads (Cat# 10004D, Thermo Fisher Scientific) and incubating for 1 h at 4°C with gentle agitation. The supernatant was collected using a magnet to discard the bead pellet. For immunoprecipitation, 8 μg of primary antibody (Mouse Monoclonal Anti-HMGB1, Cat# MAB1690, R&D Systems, USA; Rabbit Polyclonal Anti-HMGB1, Cat# ab18256, Abcam, UK; HA-Tag Rabbit mAb, Cat# AE105, ABclonal, China) or IgG control (Rabbit control IgG, Cat# ab37415, Abcam; Mouse Control IgG, Cat# AC011, ABclonal) was added to the precleared lysates (8 μg antibody per 1 mg protein) and incubated overnight at 4°C on a rotator. Separately, 50 μl of bead slurry was equilibrated in lysis buffer with protease inhibitors, washed three times with 5% BSA in PBS, and resuspended in 150 μl of lysis buffer. These beads were added to the protein-antibody complexes and incubated overnight at 4°C. The beads were then pelleted using a magnet and washed five times with 500 μl of ice-cold lysis buffer. Finally, 50 μl of 1x Laemmli sample buffer, diluted with lysis buffer, was added to the beads, vortexed, and heated at 95°C for 10 min, and the supernatant was collected for western blotting.

### 4.16 Immunoblot

Human lung tissue protein was extracted using the AllPrep DNA/RNA/Protein Mini Kit. Mice lung tissues were homogenized in 1 mL of RIPA lysis buffer (Cat# P0013C, Beyotime, China) with steel beads, supplemented with EDTA-Free Protease Inhibitor Cocktail, and centrifuged at 14,000g for 20 min at 4°C to eliminate cell debris. Similarly, pellets from HPMECs were lysed in 100 μL of RIPA lysis buffer. Protein concentrations were determined using the BCA assay (Enhanced BCA Protein Assay Kit, Cat# P0010, Beyotime, China) following the manufacturer's instructions. For analysis, 50 μg of protein was loaded onto BeyoGel™ Plus PAGE gels (Tris-Gly, 4-20%, Cat# P0468 M/P0469 M, Beyotime, China) and transferred onto Immobilon®-NC Transfer membranes (Cat# HATF00010, Millipore, USA) using the Trans-Blot Turbo Transfer System (Bio-Rad, USA). Membranes were blocked with 5% non-fat milk for 1 h at room temperature, followed by overnight incubation with primary antibodies at 4°C. The membranes were then treated with horseradish peroxidase-conjugated secondary antibodies for 1 h at room temperature. Chemiluminescence signals were detected using the MiniChemi™ 610 Mini Chemiluminescent Imaging and Analysis System (Sinsage, China). The antibodies used are listed in Table 4.

In certain experiments (Fig. 1f), S-nitrosylated protein detection was carried out using the Pierce™ S-Nitrosylation Western Blot Kit (Cat#90105, Thermo), following the manufacturer's instructions. Briefly, lung lysates were prepared under non-reducing conditions. Free cysteine thiols were blocked with MMTS, and S-nitrosylated cysteines were selectively reduced with sodium ascorbate. The newly exposed thiols were labeled with iodo-TMTzero reagent, and labeled proteins were resolved by SDS–PAGE and detected by Western blotting using an anti-TMT antibody.

### 4.17 Histology

In some experiments, the left lung was fixed by inflation using 10% formalin, dehydrated by ethanol, embedded in paraffin, and cut into 5 μm sections for analysis of histology. Tissue sections were stained with H&E stain kit (Cat# G1005, Servicebio, China) or Masson's trichrome stain kit (Cat# G1006, Servicebio). The images were captured with Evident Slidescanner Slideview VS200 (Olympus, Japan). Images were initially analyzed using OlyVIA 4.1 software (Olympus, Japan) and further processed and analyzed with ImageJ (National Institutes of Health, USA).

### 4.18 Immunofluorescence staining

Formalin fixed, paraffin embedded lung tissue sections underwent antigen retrieval with Tris-EDTA Buffer (pH = 9.0, 10 mM Tris Base, 1 mM EDTA) and then were blocked with normal donkey serum (Cat# SL050, Solarbio, China) for 1 h at room temperature, incubated with 1/1000 dilution of primary antibody (Rabbit Recombinant Monoclonal Anti-CD31 (PECAM1) (Cat# ab182981, Abcam), Mouse Monoclonal Anti-GSNOR (Cat# 66193-1-Ig, Proteintech), Rabbit Polyclonal Anti-HMGB1 (Cat# ab18256, Abcam), Mouse Monoclonal Anti-HMGB1 (Cat# MAB1690, R&D Systems), Rabbit Polyclonal Anti-HA tag (Cat# ab9110, Abcam), Mouse Monoclonal Anti-Actin, α-smooth Muscle (Cat# A2547, Sigma-Aldrich)) at 4^°^C overnight, followed by incubation with 1/500 dilution of secondary antibody (Donkey Anti-mouse-Alexa fluor 488 (Cat# ab150105, Abcam), Donkey Anti-Rabbit-Alexa fluor 555 (Cat# ab150074, Abcam), Donkey Anti-Rabbit-Alexa fluor 647 (Cat# ab150155, Abcam)) for 1 h at room temperature. Specifically, to enhance the detection of tdTomato in EC lineage-tracing mice, a rat anti-RFP antibody (Cat# 5F8, Proteintech) was used, followed by a secondary Donkey anti-Rat Alexa Fluor 555 antibody (Cat# ab150054, Abcam). 1 μg/mL DAPI (Cat # 10236276001, Roche) was used for nuclear counterstaining. ProLong™ Diamond Antifade Mountant with DAPI (Cat #P36930, Invitrogen) was used as mounting media. Images were captured with Evident Slidescanner Slideview VS200 (Olympus, Japan) or acquired by ZEISS LSM 980 with Airyscan 2 confocal microscope (Carl Zeiss Inc.), using a Plan Apochromatic 40X (NA 1.3) oil objective (Carl Zeiss), and by ZEN 3.2 system (blue edition) software. Images were further processed and analyzed using ImageJ (NIH).

### 4.19 RNA-sequencing (RNA-seq) and analysis

Total RNA was isolated using RNeasy Plus Mini Kit (Cat# 74134, QIAGEN). All samples showed an RNA integrity number (RIN) > 8. RNA-seq libraries were prepared using the NEBNext® Ultra™ RNA Library Prep Kit for Illumina® (Cat# E7530L, NEB, USA). Samples were prepared according to the library kit manufacturer's protocol, indexed, pooled, and sequenced on an Illumina NovaSeq 6000 (paired-end 150 bp). RNA sequencing was performed by Annoroad Gene Technology Co., Ltd. RNA-seq reads were aligned to the mouse reference genome GRCm38 using HISTAT2 (v2.1.0) [46]. Reads quantification for each gene was performed using HTSeq (v0.6.0) [47]. Heatmap for gene expression was created using R.

### 4.20 Lung tissue proteins preparation for proteomics

For regular quantitative proteomics of total proteins that are used as internal control of SNO-protein, lung tissues were ground into powder in liquid nitrogen and transferred to 5 mL tubes. Lysis buffer containing 1% SDS and 1% protease inhibitor cocktail was added, followed by 3 min of sonication on ice. Tissue debris was removed by centrifugation at 12,000 g for 10 min at 4°C, and protein concentrations were determined using a BCA assay. Proteins were precipitated with pre-cooled acetone, redissolved in 200 mM triethylammonium bicarbonate buffer, and digested with trypsin at a 1:50 enzyme-to-protein mass ratio overnight. The samples were then reduced with 5 mM dithiothreitol for 60 min at 37°C and alkylated with 11 mM iodoacetamide for 45 min at room temperature in the dark. Trypsin was then added at a ratio of 1:100 (proteinase: protein, m/m) and incubated for 4h at 37°C. Tryptic peptides were desalted using a Strata X SPE column (Phenomenex, USA), labeled with Tandem Mass Tag (TMT) reagents (Thermo Fisher Scientific) according to the manufacturer's instructions, pooled, desalted, and analyzed by LC–MS/MS.

For S-nitrosylation proteomics, a separate aliquot of lung tissue was ground into powder in liquid nitrogen and lysed in a buffer containing 1% SDS, 1% protease inhibitor cocktail, and 25 mM iodoacetamide (IAM), then sonicated on ice for 3 min. Tissue debris was removed by centrifugation at 12,000 g for 10 min at 4°C. The supernatant was transferred to a new tube, supplemented with an additional 25 mM IAM, and incubated for 1 h at room temperature in the dark to block free cysteine thiols. Protein concentrations were then determined using a BCA assay. Then protein S-nitrosylation analysis was performed using the iodo-TMT-based workflow with the iodo-TMT sixplex Kit (Cat. No. 90102, Thermo Fisher Scientific). Briefly, equal amounts of protein from each group were adjusted to the same volume with lysis buffer. S-nitrosylated cysteines were selectively reduced with 10 mM ascorbic acid at 37°C for 1 h in the dark. Subsequently, iodo-TMT reagents were added, and the samples were incubated in the dark at 37°C for 1 h for labeling. After labeling, dithiothreitol (DTT) was supplemented to a final concentration of 20 mM, and the mixture was incubated for 15 min in the dark.

Proteins were precipitated with pre-cooled acetone, redissolved in 200 mM triethylammonium bicarbonate buffer, and digested with trypsin at a 1:50 enzyme-to-protein mass ratio overnight. Following digestion, the samples were sequentially reduced with 5 mM DTT at 37°C for 60 min and alkylated with 11 mM iodoacetamide at room temperature for 45 min in the dark. Trypsin was then added at a ratio of 1:100 (proteinase: protein, m/m) and hydrolyze for 4h at 37°C. Finally, peptide desalting was carried out using a Strata X SPE column.

For enrichment of SNO-derived peptides, the iodo-TMT-labeled peptides were dissolved in enrichment buffer (1× TBS) and incubated with pre-washed anti-TMT resin overnight at 4°C with gentle rotation. The resin was washed sequentially with enrichment buffer and deionized water. Bound peptides were eluted with 50% acetonitrile/0.4% trifluoroacetic acid, dried by vacuum centrifugation, desalted using C18 ZipTips (Millipore), dried again, and subjected to LC–MS/MS analysis.

### 4.21 LC-MS/MS analysis

The tryptic peptides were separated on an EASY-nLC 1200 UPLC system (ThermoFisher Scientific). The separated peptides were analyzed using an Orbitrap Exploris 480 with a nano-electrospray ion source. The MS scan range was 400-1200 *m*/*z* at a resolution of 60,000, with MS/MS scans at a resolution of 15,000. Up to 25 of the most abundant precursors were selected for MS/MS analysis with a 30 s dynamic exclusion, using HCD fragmentation at a normalized collision energy of 35%. LC-MS/MS was performed by Jingjie PTM BioLab (Hangzhou) Co., Inc. The resulting MS/MS data were processed using MaxQuant search engine (v.1.6.15.0). Tandem mass spectra were searched against Mus_musculus_10090_SP_20230103.fasta (17132 entries) concatenated with reverse decoy and contaminants database. Trypsin/P was specified as cleavage enzyme allowing up to 2 missing cleavages. Min. peptide length was set as 7 and max. number of modification per peptide was set as 5. The mass tolerance for precursor ions was set as 20 ppm in first search and 20 ppm in main search, and the mass tolerance for fragment ions was set as 20 ppm. Carbamidomethyl on Cys was specified as fixed modification. Acetylation on protein N-terminal, oxidation on Met and S-nitrosylation were specified as variable modifications. False discovery rate (FDR) of protein, peptide and PSM was adjusted to < 1%.

### 4.22 Bioinformatic analysis of proteomics

For total proteome analysis, TMT-labeled peptides were identified by database searching, and total protein abundance was quantified using TMT reporter ion intensities. For S-nitrosylation proteomics, iodo-TMT-labeled SNO-site peptides were identified by database searching, and SNO-site abundance was quantified using iodo-TMT reporter ion intensities. The relative quantification of proteins was calculated based on signal intensity values (Reporter Intensity) of peptides in different samples. First, peptide signal intensities (I) were centralized to obtain relative quantification values (U) using the formula U_ij_ = I_ij_/Mean (Ij), where i represents the sample, and j represents the peptide. These values were normalized using median normalization (NR), calculated as NRij = Uij/Median (Ui) to correct for systematic errors in mass spectrometry sample loading. Finally, the relative protein quantification (R) was determined as the median of the NR values of its specific peptides, using the formula Rik = Median (NRij, j ∈ k), where k represents the protein, and j represents the specific peptides belonging to the protein. For SNO proteomics analysis, to eliminate the influence of protein expression on modification levels, the relative quantification of each SNO site was normalized by dividing it by the corresponding protein's relative quantification. Normalized SNO ratios were then used for downstream differential S-nitrosylation analysis.

The fold change (FC) between two sample groups (A and B) is calculated as the ratio of the mean relative quantification values of a protein in each group: FC_A/B,k_ = Mean (R_ik_,i ∈ A)/Mean (R_ik_,i ∈ B). To assess significance, a T-test is performed on the log2-transformed relative quantification values, with the P value indicating significance (P < 0.05): P_k_ = T.test (Log2 (R_ik_,i ∈ A), Log2 (R_ik_,i ∈ B)). Based on the differential analysis, a P value < 0.05 was considered significant. A fold change > 1.1 was used as the threshold for upregulation and < 1/1.1 for downregulation. Enrichment analysis was performed using ShinyGO [48]. Heatmaps were created using R.

### 4.23 Public scRNA-seq data analysis

To explore the expression of the *ADH5* gene in human lung endothelial subtypes, we used two public human scRNA-seq datasets (GSE128033 ^17^ and GSE136831 ^16^) from healthy and IPF lungs. Raw count matrix files were downloaded from Gene Expression Omnibus (https://www.ncbi.nlm.nih.gov/geo/). Subsequently, we used the Seurat (v4.3.0) [49] standard pipeline to perform the following analysis: quality control, dimension reduction, cell clustering, and visualization using uniform manifold approximation and projection. Harmony was used for batch correction. For GSE128033, we first annotated all the major cell types, including immune cells, stromal cells (ECs, fibroblasts, smooth muscle cells, and pericytes), and epithelial cells using canonical markers. Then, ECs were extracted to perform sub-clustering. Finally, cells were annotated with canonical markers for different endothelial subtypes. For GSE136831, we extracted the ECs according to the downloaded annotation file and then performed sub-clustering analysis. Cells were annotated according to the raw annotations. Wilcoxon rank sum test was used to compare the expression of the *ADH5* gene for different endothelial subtypes between healthy controls and IPF patients. In particular, only samples for each endothelial subtype with more than 5 cells were retained for the statistical test.

### 4.24 Enrichment analysis of amino acids flanking cysteine-SNO sites

To analyze the amino acid composition surrounding the cysteine-SNO sites, we downloaded the sequences of all the proteins detected in our murine lung tissue using mass spectrometry from the UniProt database (https://www.uniprot.org/). First, the overall frequency of each amino acid was calculated for all the detected proteins, SNO-proteins, and GR-SNO-proteins, respectively. We then extracted the flanking 10 bp protein sequences of each cysteine-SNO site, and the frequency of each amino acid for each flanking site was calculated. Finally, amino acid enrichment scores of all the flanking sites were calculated as the ratio of the frequency of SNO-proteins or SNO-down-proteins to that of all the proteins. Enrichment results were visualized using the R pheatmap package (https://github.com/raivokolde/pheatmap, v1.0.12). Motif sequences flanking the cysteine-SNO sites were analyzed using the R ggseqlogo package (v0.2) [50].

### 4.25 Statistics

For statistical analyses, we used Prism 10.0 (GraphPad Software Inc, San Diego, CA). Statistical significance was assessed using the Student's t-test or one-way ANOVA as indicated. A P value < 0.05 was considered statistically significant.

## Data availability

Bulk RNA-seq data generated during this study are deposited in the NCBI Gene Expression Omnibus (GEO) under accession number GSE289939. The mass spectrometry proteomics data have been deposited to the ProteomeXchange Consortium via the PRIDE [51] partner repository with the dataset identifier PXD061250.

## CRediT authorship contribution statement

**Zhimin Song:** Conceptualization, Data curation, Formal analysis, Funding acquisition, Investigation, Methodology, Project administration, Software, Supervision, Validation, Visualization, Writing – original draft. **Yun Zhang:** Data curation, Formal analysis, Investigation, Methodology, Software, Validation, Visualization. **Jingjing Chen:** Data curation, Formal analysis, Methodology, Software, Validation, Visualization. **Xing Peng:** Data curation, Formal analysis, Investigation, Methodology, Software, Validation, Visualization. **Jiaying Fan:** Data curation, Investigation, Methodology. **Yao Pan:** Data curation, Methodology. **Xiuxia Yang:** Methodology. **Tinghong Zhang:** Methodology. **Hui Zheng:** Methodology. **Shu Xia:** Resources. **Qun Luo:** Resources. **Bin Zhou:** Resources. **Shu Meng:** Conceptualization, Funding acquisition, Project administration, Supervision, Writing – review & editing.

## Declaration of competing interest

S.M., Z.S., Y.Z., J.C., and T.Z. hold a patent related to this work based on this study. Other authors declare no competing interests.
