# Supplementary material for: GSNOR reprograms nitrosylation to drive endothelial-to-mesenchymal transition and fibrotic vascular remodeling
Source: Redox Biol. 2026 Aug 7;96:104339. doi: 10.1016/j.redox.2026.104339 (PMC13487625; doi:10.1016/j.redox.2026.104339)
Supplement: Supplementary file 1 [file mmc1.docx]

**Supplementary Materials**

**Figure S1: Global protein S-nitrosylation changes after bleomycin-induced lung injury.**

(a) Western blot analysis of global protein S-nitrosylation levels in lung tissue from mice at day 0 and day 21 after BLM challenge, detected using an anti-S-nitrosylation antibody. S-nitrosylated protein signals, particularly those above 70 kDa, were decreased on day 21 after BLM challenge. β-actin was used as the loading control. n = 3 mice per group.

**Figure S2: EndoMT marker expression in mouse models and human IPF tissues.**

(a-b) GSNOR expression in ECs of IPF patients compared to non-IPF donors, based on data from (a) GSE136831 and (b) GSE128033, IPF Cell Atlas.

(c) qPCR analysis showed increased *Snai1*, *Acta2*, *Col1a1*, and *Fn1* gene expression in BLM-treated mice at 14 and 21 days. n ≥ 3 mice per group, mean ± SEM. *P < 0.05, **P < 0.01, ***P < 0.001, one-way ANOVA.

(d) Representative Western blots for SNAIl, α-SMA, and PECAM1 in lung homogenates from BLM-treated mice at 21 days. These proteins were detected on the same membrane using the same set of samples as shown in Fig. 1b; therefore, the same β-actin loading control is shown. n = 3 mice per group.

(e) Densitometric analysis of SNAIl, α-SMA, and PECAM1 protein levels. n = 3 mice per group, mean ± SEM. **P < 0.01, Student's t-test.

(f) qPCR analysis showed increased *Adh5*, *Snai1*, and *Col1a1* gene expression in isolated tdTomato⁺ cells from PBS- or BLM-treated EC lineage tracing mice (*Cdh5*-creERT2; *R26R*-tdTomato) at 14 days. n = 3 mice per group, mean ± SEM. *P < 0.05, **P < 0.01, Student's t-test.

(g) Densitometric analysis of Western blots, showing significant differences in protein levels between IPF patients and non-IPF donors. n = 3 independent human samples per group, mean ± SEM. **P < 0.01, Student's t-test.

**Figure S3: TGF-β2-Induced EndoMT and *ADH5* knockdown in HPMECs.**

(a) qPCR analysis in HPMECs stimulated with 10 ng/mL TGF-β2, showing significant increases in *SNAI1* and *ACTA2* expression at 24 hours, and significant increases in *SNAI1*, *SM22*, *VIM*, *COL1A1*, *FN1*, and *HMGB1* expression at 72 hours. *PECAM1* expression was slightly reduced at 72 hours. Data are presented as mean ± SEM; n ≥ 3 independent biological replicates per group. *P < 0.05, **P < 0.01, ***P < 0.001, ****P < 0.0001. One-way ANOVA was used for *SNAI1* and *ACTA2* across multiple time points; Student’s t-test was used for genes with two-group comparisons.

(b-e) Western blot and densitometric analysis of HPMECs stimulated with 10 ng/mL TGF-β2 at 6 and 24 hours. Representative blots and quantification bar plots showing increased levels of SNAI1, FN, and HMGB1 at both 6 and 24 hours, PECAM1 upregulation at 24 hours, and CD144 upregulation at both 6 and 24 hours. No significant change in α-SMA protein levels was observed between control and TGF-β2 stimulated groups. Data are presented as mean ± SEM; n ≥ 3 independent biological replicates per group, except for CD144 at 6 h, where n = 2. *P < 0.05, **P < 0.01, Student's t-test.

(f) Knockdown efficiency of *ADH5* in HPMECs using siRNA, showing significant reduction in *ADH5* mRNA levels at 48 hours. n = 5 independent biological replicates per group, mean ± SEM. ****P < 0.0001, Student's t-test.

(g-h) Western blot analysis showing decreased GSNOR protein levels in *ADH5* knockdown HPMECs at 48 hours. Representative blots and densitometric analysis are shown. n = 4 independent biological replicates per group, mean ± SEM. ***P < 0.001, Student's t-test.

**Figure S4: Pharmacological inhibition of GSNOR by N6022 attenuates leukocytes infiltration in the lung.**

(a) Schematic of the experimental design: WT mice were administered 1 mg/kg and 30 mg/kg of the GSNOR-specific inhibitor N6022 intraperitoneally one day prior to a 5 U/kg BLM challenge. Samples were collected after 14 days.

(b) The representative gating strategy for identifying cell populations from bronchoalveolar lavage (BAL) fluid by flow cytometry is based on specific surface markers. T cells were gated as CD45⁺ CD11b⁻ CD3⁺, while B cells were identified as CD45⁺ CD11b⁻ B220⁺. Eosinophils and alveolar macrophages were distinguished as CD45⁺ CD11c⁻ SiglecF⁺ and CD45⁺ CD11c⁺ SiglecF⁺, respectively. Proinflammatory monocytes were gated as CD45⁺CD11b⁺SiglecF⁻ Ly6C^high^Ly6G⁻, and neutrophils were identified as CD45⁺CD11b⁺SiglecF⁻Ly6C^int^Ly6G⁺.

(c) Flow cytometry analysis of cell composition in BAL fluid.

(d) Total leukocyte counts from 3 mL BAL fluid followed by differential cell counts analyzed by flow cytometry.

(c-d) n ≥ 3 mice per group, mean ± SEM. *P < 0.05, **P < 0.01, ***P < 0.001, ****P < 0.0001, one-way ANOVA.

**Figure S5: Pharmacological inhibition of GSNOR by N91115 mitigates EndoMT and pulmonary fibrosis.**

(a) Schematic of the experimental design: WT mice were administered 1 mg/kg, 5mg/kg, or 10 mg/kg of the GSNOR-specific inhibitor N91115 orally one day prior to a 5 U/kg BLM challenge. N91115 was then given orally every two days post-challenge. Samples were collected after 14 days.

(b) Flow cytometry analysis of cell composition in BAL fluid.

(c) Total leukocyte counts from 3 mL BAL fluid followed by differential cell counts analyzed by flow cytometry. (b-c) n ≥ 6 mice per group, means ± SEM. *P < 0.05, **P < 0.01, one-way ANOVA.

(d) Masson's trichrome staining of lung sections showing reduced collagen deposition in 10 mg/kg N91115-treated mice compared to vehicle controls challenged with BLM.

(e) Quantification of collagen area relative to total lung area in vehicle controls and 10 mg/kg N91115-treated mice, both challenged with BLM. n ≥ 5 mice per group, mean ± SEM. ***P < 0.001, Student's t-test.

(f) Quantification of hydroxyproline levels in lung tissue using hydroxyproline assay kits, indicating reduced collagen content in 10 mg/kg N91115-treated mice compared to vehicle controls challenged with BLM. n ≥ 3 mice per group, mean ± SEM. *P < 0.05, Student's t-test.

(g) Western blot analysis showing decreased SNAI and α-SMA expression and increased CD144 expression in 10 mg/kg N91115-treated mice compared to vehicle controls challenged with BLM. n = 3 mice per group.

(h) Densitometric analysis of GSNOR, SNAI, CD144, and α-SMA protein levels. n = 3 mice per group, mean ± SEM. *P < 0.05, **P < 0.01, ***P < 0.001, one-way ANOVA.

**Figure S6: Genetic deletion of *Adh5* reduces EndoMT and pulmonary fibrosis in mice.**

(a) Representative PCR genotyping results distinguishing *Adh5* wild-type (WT), heterozygous (HE), and homozygous knockout (KO) mice.

(b) Nucleotide sequences of the WT and KO alleles, highlighting the genomic alterations in *Adh5* KO mice.

(c) Schematic of the experimental design: *Adh5* KO and WT mice were challenged with 2.5 U/kg BLM intratracheally, and samples were collected 21 days post-BLM challenge.

(d) Western blot analysis showing decreased SNAI1 in *Adh5* KO mice compared to WT controls at 21 days post-BLM challenge. n = 3 mice per group.

(e) Densitometric analysis of GSNOR, SNAI, CD144, and α-SMA protein levels. n = 3 mice per group, mean ± SEM. *P < 0.05, **P < 0.01, ***P < 0.001, one-way ANOVA.

(f) Masson's trichrome staining of lung sections showed reduced collagen deposition in *Adh5* KO mice compared to WT controls at 21 days post-BLM challenge.

(g) Quantification of collagen area relative to total lung area in WT and *Adh5* KO mice, both challenged with BLM. n ≥ 5 mice per group, mean ± SEM. *P < 0.05, Student's t-test.

(h) Quantification of hydroxyproline levels in lung tissue using hydroxyproline assay kits, indicating reduced collagen content in *Adh5* KO mice compared to WT controls challenged with BLM at 21 days. n ≥ 3 mice per group, mean ± SEM. ***P < 0.001, ****P < 0.0001, one-way ANOVA.

(i) Flow cytometry analysis of cell composition in BAL fluid.

(j) Total leukocyte counts from 3 mL BAL fluid followed by differential cell counts analyzed by flow cytometry.

(i-j) n ≥ 4 mice per group, except for the *Adh5* KO PBS group, where n = 2, mean ± SEM. *P < 0.05, **P < 0.01, one-way ANOVA.

**Figure S7: EC-Specific deletion of *Adh5* attenuates leukocytes infiltration in the Lung.**

(a) Representative PCR genotyping results distinguishing WT and *Adh5*^fl/fl^ mice.

(b) Nucleotide sequences of the WT and *Adh5*^fl/fl^ alleles, highlighting the genomic alterations in *Adh5*^fl/fl^ mice.

(c) Flow cytometry analysis of cell composition in BAL fluid at 14 days post-BLM challenge.

(d) Total leukocyte counts from 3 mL BAL fluid followed by differential cell counts analyzed by flow cytometry.

(c-d) n ≥ 4 mice per group, mean ± SEM. *P < 0.05, **P < 0.01, ***P < 0.001, one-way ANOVA.

**Figure S8: EC-Specific *Adh5* overexpression aggravates leukocyte infiltration.**

(a) Representative PCR genotyping results distinguishing *Rosa26 ^+/+^* (WT), and heterozygous *Rosa26* ^LSL^*^-Adh5/+^* (HE) and homozygous *Rosa26* ^LSL^*^-Adh5/^* ^LSL^*^-Adh5^* (OE) mice.

(b) Nucleotide sequences of the WT and OE alleles, highlighting the genomic alterations in *Rosa26* ^LSL^*^-Adh5^* mice.

(c) Flow cytometry analysis of cell composition in BAL fluid at 14 days post-BLM challenge.

(d) Differential cell counts were obtained from 3 mL of BAL fluid and analyzed by flow cytometry.

(c-d) n ≥ 5 mice per group, except for the Ctrl PBS group, where n = 2, mean ± SEM.

**Figure S9. Amino acid composition and flanking sequence features of SNO and GR-SNO sites.**

(a) Amino acid composition of whole lung, SNO, and GR-SNO proteins.

(b) Sequence logo depicting amino acid composition flanking cysteines (±10 residues) in whole lung proteins. Letter size indicates the relative frequency of each amino acid at the corresponding position.

(c) Sequence logo showing the amino acid composition flanking SNO sites (±10 positions surrounding validated S-nitrosylated cysteines). Letter size represents the relative frequency of each amino acid at the corresponding position.

(d) Amino acid fold enrichment at each position flanking SNO cysteines. Fold enrichment was calculated as the ratio of amino acid composition at each of the ±10 positions surrounding SNO cysteines to the overall amino acid composition in whole lung proteins.

(e) Percentage of charged amino acid motifs flanking cysteine in the whole lung, SNO, and GR-SNO proteins. The analyzed motifs include A-C-A, B-C-B, A-C-B, B-C-A, A-C-X, B-C-X, X-C-A, X-C-B, and X-C-X, where **A** represents an acidic amino acid, **B** a basic amino acid, **C** cysteine, and **X** a non-charged amino acid. In whole lung proteins, percentages reflect the occurrence of these motifs among all cysteines. In SNO and GR-SNO proteins, percentages are calculated based only on sequences flanking experimentally validated S-nitrosylation sites.

(f) Tables displaying selected GR-SNO sites where cysteines are flanked by charged amino acids in -1 or/and +1 positions.

**Figure S10: Knockdown of *HMGB1* and overexpression of *HMGB1* in HPMECs.**

(a) Knockdown efficiency of *HMGB1* in HPMECs using siRNA, showing significant reduction in *HMGB1* mRNA levels at 48 hours. n = 5 independent biological replicates per group, mean ± SEM. ****P < 0.0001, Student's t-test.

(b) Western blot analysis confirming decreased HMGB1 protein levels in *HMGB1* knockdown HPMECs at 48 hours. Representative blots and densitometric analysis are shown. n = 3 independent biological replicates per group, mean ± SEM. ***P < 0.001, Student's t-test.

(c) Sequence analysis showing that the cysteine residues are conserved across species.

(d) Schematic representation of the construction of HA-tagged HMGB1(WT) and HMGB1(C23A) plasmids.

(e) Sequence showing the mutation of Cysteine 23 to Alanine.

(f) qPCR analysis in HPMECs transfected with HA-tagged HMGB1(WT) and HMGB1(C23A) constructs, showing successful overexpression of both constructs. n = 3 independent biological replicates per group, mean ± SEM. ***P < 0.001, one-way ANOVA.

(g) Western blot analysis confirming successful overexpression of HA-tagged HMGB1(WT) and HMGB1(C23A) constructs in HPMECs. Representative blots are shown.
