# Supplementary material for: GSNOR reprograms nitrosylation to drive endothelial-to-mesenchymal transition and fibrotic vascular remodeling
Source: Redox Biol. 2026 Aug 7;96:104339. doi: 10.1016/j.redox.2026.104339 (PMC13487625; doi:10.1016/j.redox.2026.104339)
Supplement: Supplementary file 2 [file mmc2.docx]

**Table 1. sgRNAs utilized in CRISPR/Cas9-mediated mouse models.**

| Mouse | gRNAs | Sequence (5’-3’) |
| --- | --- | --- |
| Adh5-KO | sgRNA1 | CATTGTTTCTTGGAGATGCC**TGG** |
|  | sgRNA2 | TTAGAGACACATCGGTGAGT**CGG** |
| Adh5-CKO | sgRNA1 | ACAAGTACAGGTAAAGCCGG**TGG** |
|  | sgRNA2 | AGTCTGGAAAGGCAACCTCA**AGG** |
| Adh5-oe | sgRNA | GGGGACACACTAAGGGAGCT**TGG** |

**Table 2. siRNA sequences.**

| **Name** | **Passenger strand** | **Guide strand** |
| --- | --- | --- |
| ADH5 siRNA#1 | AAUUCAGUCAUGAACCUGUUUUC**dTdG** | CAGAAAACAGGUUCAUGACUGAAUUAU |
| ADH5 siRNA#2 | GUGUCUGAAUAUAUGUCCAAAAA**dGdA** | UCUUUUUGGACAUAUAUUCAGACACCA |
| ADH5 siRNA#3 | UCCUUUGAAUGUAUUGGUAAUGU**dGdA** | UCACAUUACCAAUACAUUCAAAGGAAU |
| HMGB1 siRNA#1 | GGAGAGATGTGGAATAACA**dTdT** | UGUUAUUCCACAUCUCUCC**dTdT** |
| HMGB1 siRNA#2 | CATGTCTGCTAAAGAGAAA**dTdT** | UUUCUCUUUAGCAGACAUG**dTdT** |
| HMGB1 siRNA#3 | CTGCGAAGCTGAAGGAAAA**dTdT** | UUUUCCUUCAGCUUCGCAG**dTdT** |

**Table 3. qPCR primer sequences.**

| **Species** | **Gene** | **Forward Sequence** | **Reverse Sequence** |
| --- | --- | --- | --- |
| Human | ADH5 | 5'-CATTGCCACTGCGGTTTGCCAC | 5'-AGTGTCACCCGCCTTCAGCTTAGT |
|  | HMGB1 | 5'-TCGGCCTTCTTCCTCTTCT | 5'-CCACATCTCTCCCAGTTTCTTC |
|  | FN1 | 5'-ACAACACCGAGGTGACTGAGAC | 5'-GGACACAACGATGCTTCCTGAG |
|  | SNAI1 | 5'-CTCTAATCCAGAGTTTACCTTC | 5'-GACAGAGTCCCAGATGAG |
|  | ACTA2 | 5'-CTATGCCTCTGGACGCACAACT | 5'-CAGATCCAGACGCATGATGGCA |
|  | VIM | 5'-AGGCAAAGCAGGAGTCCACTGA | 5'-ATCTGGCGTTCCAGGGACTCAT |
|  | SM22 | 5'-GCAGTCCAAAATCGAGAAGAAG | 5'-CAGAATCACGCCATTCTTCAG |
|  | PECAM1 | 5'-AAGTGGAGTCCAGCCGCATATC | 5'-ATGGAGCAGGACAGGTTCAGTC |
|  | CDH5 | 5'-GCGACTACCAGGACGCTTTC | 5'-GGCTTCATGGGCTTGATGAT |
|  | COL1A1 | 5'-GATTCCCTGGACCTAAAGGTGC | 5'-AGCCTCTCCATCTTTGCCAGCA |
|  | 18S rRNA | 5'-CTCAACACGGGAAACCTCAC | 5'-CGCTCCACCAACTAAGAACG |
| Mouse | Adh5 | 5'-GGCAACGTGAAGGTCATGAGA | 5'-GCTACTCCCACTACCACACTGACA |
|  | Fn1 | 5'-GGCCACCATTACTGGTCTGG | 5'-GGAAGGGTAACCAGTTGGGG |
|  | Snai1 | 5'-GCCGGAAGCCCAACTATAGC | 5'-GGTCGTAGGGCTGCTGGAA |
|  | Acta2 | 5'-GTCCCAGACATCAGGGAGTAA | 5'-TCGGATACTTCAGCGTCAGGA |
|  | Col1a1 | 5'-CGATGGATTCCCGTTCGAGT | 5'-CGATCTCGTTGGATCCCTGG |
|  | 18S rRNA | 5'-CGAAAGCATTTGCCAAGAAT | 5'-AGTCGGCATCGTTTATGGTC |

**Table 4. Antibodies used for immunoblotting.**

| **Antibody** | **Source** | **Cat#** | **Reactive Species** | **Dilution** |
| --- | --- | --- | --- | --- |
| Mouse Monoclonal Anti-S-nitrosocysteine | Abcam | ab94930 | human, mouse | 1:1000 |
| Rabbit Polyclonal Anti-GSNOR | Proteintech | 16379-1-AP | human, mouse | 1:1000 |
| Mouse Monoclonal Anti-GSNOR | Proteintech | 66193-1-Ig | human, mouse | 1:1000 |
| Mouse Monoclonal Anti-HMGB1 | R&D Systems | MAB1690 | human | 1:1000 |
| Rabbit Polyclonal Anti-HMGB1 | Abcam | ab18256 | human, mouse | 1:1000 |
| Rabbit Polyclonal Anti-HA tag | Abcam | ab9110 | NA | 1:4000 |
| Rabbit Polyclonal Anti-β-tubulin | Abclonal | AC008 | human, mouse | 1:1000 |
| Rabbit Polyclonal Anti-Histone H3 | Abcam | ab1791 | human, mouse | 1:1000 |
| Rabbit Polyclonal Anti-Anctin β | Immunoway | YT0099 | human, mouse | 1:1000 |
| Rabbit Polyclonal Anti-Fibronectin | Proteintech | 15613-1-AP | human, mouse | 1:1000 |
| Rabbit Polyclonal Anti-SNAI1 | Abclonal | A11794 | human, mouse | 1:1000 |
| Mouse Monoclonal Anti-Actin, α-smooth Muscle | Sigma | A2547 | human, mouse | 1:1000 |
| Rabbit Polyclonal Anti-Collagen Type I | Rockland | 600-401-103-0.1 | human, mouse | 1:1000 |
| Mouse Monoclonal Anti-TGF-Beta 1 | R&D Systems | MAB240 | human, mouse | 1:1000 |
| Rabbit Monoclonal Anti-Smad2/Smad3 | Abcam | ab202445 | human, mouse | 1:1000 |
| Rabbit Monoclonal Anti-Phospho-Smad2/Smad3 | Abclonal | AP1343 | human, mouse | 1:1000 |
| Mouse Monoclonal Anti-CD31 | Abcam | ab9498 | human, mouse | 1:1000 |
| Mouse Monoclonal Anti-VE-cadherin | Santa Cruz | sc9989 | human, mouse | 1:200 |
| Anti-Rabbit lgG,HRP-linked Antibody | Cell Signalling Technology | 7074S | NA | 1:2000 |
| Anti-Mouse lgG,HRP-linked Antibody | Cell Signalling Technology | 7076S | NA | 1:2000 |
